# Checklist of Fabaceae Lindley in Balaghat Ranges of Maharashtra, India

**DOI:** 10.3897/BDJ.3.e4541

**Published:** 2015-03-05

**Authors:** Ramchandra Gore, Sayajirao Gaikwad

**Affiliations:** ‡ Life Science Research Laboratory, Walchand College of Arts and Science, Solapur- 413 006, Maharashtra, India

**Keywords:** Legumes, enumeration, Balaghat Ranges, India

## Abstract

The present paper provides an enumeration of leguminous taxa of Balaghat Ranges of Maharashtra along with their habits, phenological deta and voucher specimen numbers. During the present work, a total of 123 species, 4 subspecies and 17 varieties of Fabaceae have been recorded for Balaghat Ranges of Maharashtra, of which 119 taxa are occurring in wild while 25 are under cultivation. The members of Fabaceae are dominant in herbaceous vegetation of the Balaghat Ranges. There are more species in genera like *Crotalaria* (23 taxa), *Indigofera* (16 taxa), *Alysicarpus* (14 taxa), *Vigna* (11 taxa) and *Desmodium* (8 taxa). Twelve taxa are endemic to India of which *Indigofera deccanensis* falls into Critically Endangered IUCN Red data category. The legumes of Balaghat Ranges have many actual and potential uses such as food, fodder and sources of edible oil, natural dyes, industrial lubricants, timber and medicines. About 19 leguminous taxa are wild relatives of food and fodder crops have resistance to pests and diseases, and abiotic stresses such as drought and salinity, can be used for crop improvement.

## Introduction

Fabaceae Lindl. *nom. alt.* (Papilionaceae Giseke *nom. cons.*) is the third largest family of flowering plants after Orchidaceae and Asteraceae, with approximately 483 genera and about 12,000 species mainly distributed in tropical and subtropical region of the World (Lewis et al. 2005). The family is an important in a diversity of ecosystems; members of it present and often dominant in nearly every vegetation type on earth, from tropical rain forests to deserts and alpine tundra (Wojciechowski 2003). In India, there are about 147 genera, 805 species, 33 subspecies, 155 varieties and 14 forma of legumes (Sanjappa 1991, Sanjappa 1995;Dave 2004;Chaudhary and Khan 2005; Ansari 2008; Jabbar et al. 2010; Aitawade et al. 2012; Chavan et al. 2013; Gaikwad et al. 2014d​). The higher concentration of legumes are in peninsular India (about 619 species) and northeast India including eastern Himalaya (about 767 species) corresponding to two hotspot areas in India. About 83 genera, 326 species, 2 subspecies, 57 varieties and 1 forma of Fabaceae occur in Maharashtra (Almeida 1998; Kothari 2000; Naik 1998; Aitawade et al. 2012; Gaikwad et al. 2012; Chavan et al. 2013; Gaikwad et al. 2014a, Gaikwad et al. 2014c, Gaikwad et al. 2014d).

A hilly terrain and semi-arid general climate of Balaghat Ranges supports unique tropical dry deciduous forests and thorny scrub vegetation with vast grasslands. The grasslands of Balaghat Ranges are unique and popularly known as Indian Savannas. The herbaceous vegetation of Balaghat Ranges is notable as it amounts to 59% of the whole flora. The members of the Fabaceae are dominant in herbaceous vegetation. The genera like *Alysicarpus* Neck. ex Desv., *Crotalaria* L., *Indigofera* L., *Desmodium* Desv. and *Vigna* Savi are most diversified in the Balaghat region. In spite of rich diversity, the legumes of Balaghat Ranges remained neglected or ignored by earlier workers probably due to the general dry climate of the region. However, some sporadic efforts have been made to study the legumes of Balaghat in the last hundred years such as Jain (1961), Jagtap (1965), Naik (1966), Bhagvat (1968), Naik (1969), Naik (1970), Wadood Khan (1982), Mahabale (1987) and Naik (1998). Thus, comprehensive and reliable account of legumes of Balaghat Ranges does not exist. Hence, the present study was undertaken in 2009 as Ph.D. work to produce an up-to-date account of the legumes of Balaghat Ranges.

## Materials and methods

### Study area

**Figure 1.**
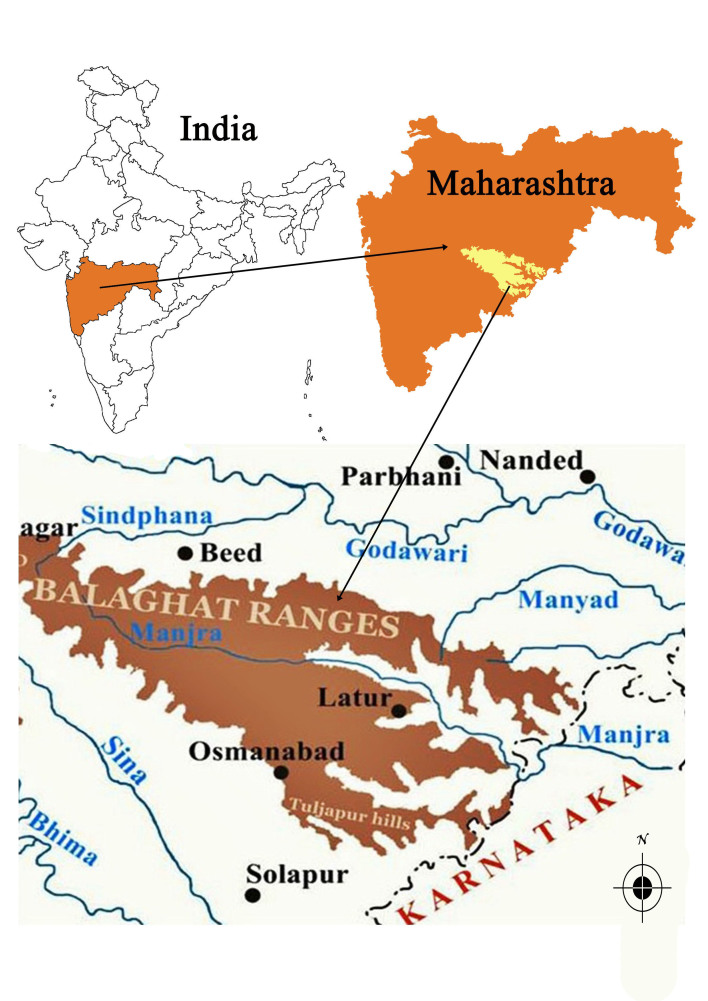
Location map of Balaghat Ranges of Maharashtra (India).

Balaghat Ranges are eastern spur of Western Ghats of India lie in between 18°47'40.26" - 18°32'29.28"N latitude and 75°20'26.90" - 76°48'56.18"E longitude, spread in Ahmednagar, Beed, Latur, Osmanabad and Solapur districts of the Maharashtra State of India in the basins of river Manjra and its tributaries Bori, Lendi, Manyad, Terna and Tiru. Balaghat occupies an area of about 18,111.34 Km^2^ (Fig. 1). The terrain of Balaghat shows hills and hillocks of varying heights (450 to 850 m), which support unique tropical dry deciduous forests, open scrub jungles and vast grasslands. The weather in general is dry and said to be moderately extreme. An annual temperature ranges between 27.7^o^C - 42^o^C and the relative humidity is extremely low (35-50%) for major part of the year while it is highest (75%) during the monsoon season (June-October). The average annual rainfall is about 729 mm mainly from June to October.

### Data collection

First of all a list of taxa of the family Fabaceae of the Balaghat Ranges was prepared by referring to all available literature and specimens deposited in various herbaria. After that, field-visits of 1-3 days duration were undertaken to nooks and corners of Balaghat Ranges for plant collection. During the field visits, information on habit, habitat, phenological data, GPS data and present status were gathered. Three specimens were collected of each species and prepared voucher specimens following standard herbarium techniques (Rao and Sharma 1990). The specimens are deposited in the herbarium of Walchand College, Solapur, Maharashtra (WACS). Identification of taxa was confirmed with the help of available literature such as Cooke (1958), Babu et al. (1987), Sanjappa (1995), Almeida (1998), Naik (1998), Kothari (2000), Lakshminarasimhan (2001), Tomooka et al. (2002a), Tomooka et al. (2002b), Dave (2004), Chaudhary and Khan (2005), Ansari (2008), Jabbar et al. (2010), Toomoka et al. (2011) and Aitawade et al. (2012). Doubtful and interesting identifications were confirmed by their direct comparison with authentically identified specimens deposited in various herbaria such as Herbarium of Botanical survey of India, Pune (BSI); Blatter Herbarium, St. Xavier’s College, Mumbai (BLAT) and BAMU Herbarium, Aurangabad (BAMUA). The botanical name of the taxa have been verified with International Plant Name Index (IPNI). The genera, species and infraspecific taxa are alphabetically arranged in the present paper. Some important plants are featured in photographs (Figs 2, 3, 4, 5).

## Checklists

### Checklist of leguminous taxa of Balaghat Ranges of Maharashtra

#### Abrus precatorius

L. 1767

##### Materials

- **Type status:** Other material. **Location:** continent: Asia; country: India; countryCode: IN; stateProvince: Maharashtra; municipality: Osmanabad; locality: Ramling; verbatimLatitude: 18°17.322N; verbatimLongitude: 75°56.637E; verbatimCoordinateSystem: degrees minutes; geodeticDatum: WGS84; **Event:** month: August-March; fieldNumber: *RDG*- 003; fieldNotes: Woody climbers; **Record Level:** institutionCode: Walchand College of Arts & Science, Solapur (WCAS).
- **Type status:** Other material. **Location:** continent: Asia; country: India; countryCode: IN; stateProvince: Maharashtra; municipality: Tuljapur; locality: Apsinga; verbatimLatitude: 18°03.982N; verbatimLongitude: 76°03.951E; verbatimCoordinateSystem: degrees minutes; geodeticDatum: WGS84; **Event:** month: August-March; fieldNumber: **​R.D.* Gore*- 13107; fieldNotes: Woody climbers; **Record Level:** institutionCode: Walchand College of Arts & Science, Solapur (WCAS).

#### Aeschynomene americana

L. 1753

##### Materials

- **Type status:** Other material. **Location:** continent: Asia; country: India; countryCode: IN; stateProvince: Maharashtra; municipality: Beed; locality: Neknur; verbatimLatitude: 18°48.504N; verbatimLongitude: 75°46.358E; verbatimCoordinateSystem: degrees minutes; geodeticDatum: WGS84; **Event:** month: October-December; fieldNumber: *RDG-* 471; fieldNotes: Erect herbs; **Record Level:** institutionCode: Walchand College of Arts & Science, Solapur (WCAS).

#### Aeschynomene indica

L. 1753

##### Materials

- **Type status:** Other material. **Location:** continent: Asia; country: India; countryCode: IN; stateProvince: Maharashtra; municipality: Ambajogai; locality: Near Talni; verbatimLatitude: 18°44.311N; verbatimLongitude: 76°30.448E; verbatimCoordinateSystem: degrees minutes; geodeticDatum: WGS84; **Event:** month: August-February; fieldNumber: *RDG-* 025; fieldNotes: Erect herbs; **Record Level:** institutionCode: Walchand College of Arts & Science, Solapur (WCAS).

#### Alhagi maurorum

Medik. 1787

##### Materials

- **Type status:** Other material. **Location:** continent: Asia; country: India; countryCode: IN; stateProvince: Maharashtra; municipality: Beed; locality: Beed; verbatimCoordinates: -; geodeticDatum: unknown; **Event:** month: March-April; fieldNumber: *V.N. Pardeshi*- 1209; fieldNotes: Shrubs; **Record Level:** institutionCode: Dr. Babasaheb Ambedkar Marathwada University, Aurangabad (BAMU).

#### Alysicarpus bupleurifoliusvar.bupleurifolius

(L.) DC. 1825

##### Materials

- **Type status:** Other material. **Location:** continent: Asia; country: India; countryCode: IN; stateProvince: Maharashtra; municipality: Osmanabad; locality: Ghatangri; verbatimLatitude: 18°13.520N; verbatimLongitude: 76°00.090E; verbatimCoordinateSystem: degrees minutes; geodeticDatum: WGS84; **Event:** month: July-September; fieldNumber: *RDG-* 005; fieldNotes: Erect herbs; **Record Level:** institutionID: Wachland College of Arts & Science, Solapur (WCAS).

#### Alysicarpus hamosus

Edgew. 1853

##### Materials

- **Type status:** Other material. **Location:** continent: Asia; country: India; countryCode: IN; stateProvince: Maharashtra; municipality: Lohara; locality: Manjra-dam; verbatimLatitude: 18°33.304N; verbatimLongitude: 76°05.868E; verbatimCoordinateSystem: degrees minutes; geodeticDatum: WGS84; **Event:** month: September-November; fieldNumber: *RDG-* 297; fieldNotes: Erect herbs; **Record Level:** institutionCode: Wachland College of Arts & Science, Solapur (WCAS).

#### Alysicarpus heyneanus

Wight & Arn. 1834

##### Materials

- **Type status:** Other material. **Location:** continent: Asia; country: India; countryCode: IN; stateProvince: Maharashtra; municipality: Patoda; locality: Lambarwadi; verbatimLatitude: 18°50.122N; verbatimLongitude: 75°19.118E; verbatimCoordinateSystem: degrees minutes; geodeticDatum: WGS84; **Event:** month: October-February; fieldNumber: *RDG-* 440; fieldNotes: Erect herbs; **Record Level:** institutionCode: Wachland College of Arts & Science, Solapur (WCAS).

#### Alysicarpus longifolius

Wight & Arn. 1834

##### Materials

- **Type status:** Other material. **Location:** continent: Asia; country: India; countryCode: IN; stateProvince: Maharashtra; municipality: Tuljapur; locality: Jalkot; verbatimLatitude: 17°49.827N; verbatimLongitude: 76°19.664E; verbatimCoordinateSystem: degrees minutes; geodeticDatum: WGS84; **Event:** month: September-January; fieldNumber: *RDG-* 353; fieldNotes: Erect herbs; **Record Level:** institutionCode: Wachland College of Arts & Science, Solapur (WCAS).

#### Alysicarpus luteo-vexillatus

Naik & Pokle, 1986

##### Materials

- **Type status:** Other material. **Location:** continent: Asia; country: India; countryCode: IN; stateProvince: Maharashtra; municipality: Osmanabad; locality: Bhanasgaon; verbatimLatitude: 18°16.839N; verbatimLongitude: 75°56.587E; verbatimCoordinateSystem: degrees minutes; geodeticDatum: WGS84; **Event:** month: September-January; fieldNumber: *RDG-* 008; fieldNotes: Erect herbs; **Record Level:** institutionCode: Wachland College of Arts & Science, Solapur (WCAS).

#### Alysicarpus monilifer

(L.) DC. 1825

##### Materials

- **Type status:** Other material. **Location:** continent: Asia; country: India; countryCode: IN; stateProvince: Maharashtra; municipality: Osmanabad; locality: Ramling; verbatimLatitude: 18°17.741N; verbatimLongitude: 75°56.712E; verbatimCoordinateSystem: degrees minutes; geodeticDatum: WGS84; **Event:** month: August-November; fieldNumber: *RDG-* 006; fieldNotes: Erect herbs; **Record Level:** institutionCode: Wachland College of Arts & Science, Solapur (WCAS).

#### Alysicarpus ovalifolius

(Schum.) Leonard, 1954

##### Materials

- **Type status:** Other material. **Location:** continent: Asia; country: India; countryCode: IN; stateProvince: Maharashtra; municipality: Patoda; locality: Khandala; verbatimLatitude: 18°51.180N; verbatimLongitude: 75°42.552E; verbatimCoordinateSystem: degrees minutes; geodeticDatum: WGS84; **Event:** month: August-November; fieldNumber: *RDG-* 1017; fieldNotes: Erect herbs; **Record Level:** institutionCode: Wachland College of Arts & Science, Solapur (WCAS).

#### Alysicarpus pubescensvar.pubescens

Law, 1840

##### Materials

- **Type status:** Other material. **Location:** continent: Asia; country: India; countryCode: IN; stateProvince: Maharashtra; municipality: Osmanabad; locality: Bhanasgaon; verbatimLatitude: 18°17.157N; verbatimLongitude: 75°56.559E; verbatimCoordinateSystem: degrees minutes; geodeticDatum: WGS84; **Event:** month: September-January; fieldNumber: *RDG-* 009; fieldNotes: Erect herbs; **Record Level:** institutionCode: Wachland College of Arts & Science, Solapur (WCAS).

#### Alysicarpus pubescensvar.vasavadae

(Hemadri) Sanjappa, 1991

##### Materials

- **Type status:** Other material. **Location:** continent: Asia; country: India; countryCode: IN; stateProvince: Maharashtra; municipality: Beed; locality: Dahiphal; verbatimLatitude: 18°49.504N; verbatimLongitude: 75°52.876E; verbatimCoordinateSystem: degrees minutes; geodeticDatum: WGS84; **Event:** month: September-January; fieldNumber: *RDG-* 381; fieldNotes: Erect herbs; **Record Level:** institutionCode: Wachland College of Arts & Science, Solapur (WCAS).

#### Alysicarpus roxburghianus

Thoth. & Pramanik, 1981

##### Materials

- **Type status:** Other material. **Location:** continent: Asia; country: India; countryCode: IN; stateProvince: Maharashtra; municipality: Ambajogai; locality: Near Ghatnandur; verbatimLatitude: 18°43.924N; verbatimLongitude: 76°31.804E; verbatimCoordinateSystem: degrees minutes; geodeticDatum: WGS84; **Event:** month: August-December; fieldNumber: *RDG-* 014; fieldNotes: Erect herbs; **Record Level:** institutionCode: Wachland College of Arts & Science, Solapur (WCAS).

#### Alysicarpus rugosus

(Willd.) DC. 1825

##### Materials

- **Type status:** Other material. **Location:** continent: Asia; country: India; countryCode: IN; stateProvince: Maharashtra; municipality: Tuljapur; locality: Tuljapur town; verbatimLatitude: 17°59.911N; verbatimLongitude: 76°04.268E; verbatimCoordinateSystem: degrees minutes; geodeticDatum: WGS84; **Event:** month: August-October; fieldNumber: *RDG-* 885; fieldNotes: Erect herbs; **Record Level:** institutionCode: Wachland College of Arts & Science, Solapur (WCAS).

#### Alysicarpus scariosus

(Rottl. ex Spreng.) Grah. ex Thw. 1859

##### Materials

- **Type status:** Other material. **Location:** continent: Asia; country: India; countryCode: IN; stateProvince: Maharashtra; municipality: Osmanabad; locality: Ramling; verbatimLatitude: 18°17.812N; verbatimLongitude: 75°56.915E; verbatimCoordinateSystem: degrees minutes; geodeticDatum: WGS84; **Event:** month: July-January; fieldNumber: *RDG-* 001; fieldNotes: Erect herbs; **Record Level:** institutionCode: Wachland College of Arts & Science, Solapur (WCAS).

#### Alysicarpus tetragonolobus

Edgew. 1853

##### Materials

- **Type status:** Other material. **Location:** continent: Asia; country: India; countryCode: IN; stateProvince: Maharashtra; municipality: Osmanabad; locality: Ghatangri; verbatimLatitude: 18°13.266N; verbatimLongitude: 75°59.915E; verbatimCoordinateSystem: degrees minutes; geodeticDatum: WGS84; **Event:** month: June-December; fieldNumber: *RDG-* 004; fieldNotes: Erect herbs; **Record Level:** institutionCode: Wachland College of Arts & Science, Solapur (WCAS).

#### Alysicarpus vaginalisvar.nummularifolius

Miq. 1855

##### Materials

- **Type status:** Other material. **Location:** continent: Asia; country: India; countryCode: IN; stateProvince: Maharashtra; municipality: Lohara; locality: Udatpur; verbatimLatitude: 17°59.188N; verbatimLongitude: 76°28.948E; verbatimCoordinateSystem: degrees minutes; geodeticDatum: WGS84; **Event:** month: August-December; fieldNumber: *RDG-* 183; fieldNotes: Erect herbs; **Record Level:** institutionCode: Wachland College of Arts & Science, Solapur (WCAS).

#### Arachis hypogaea

L. 1753

##### Materials

- **Type status:** Other material. **Location:** continent: Asia; country: India; countryCode: IN; stateProvince: Maharashtra; municipality: Omerga; locality: Wagdari; verbatimLatitude: 17°54.785N; verbatimLongitude: 76°39.982E; verbatimCoordinateSystem: degrees minutes; geodeticDatum: WGS84; **Event:** month: October-February and May-June; fieldNumber: *RDG-* 577; fieldNotes: Erect or prostrate herbs; **Record Level:** institutionCode: Wachland College of Arts & Science, Solapur (WCAS).
- **Type status:** Other material. **Location:** continent: Asia; country: India; countryCode: IN; stateProvince: Maharashtra; municipality: Tuljapur; locality: Bori (Apsinga); verbatimLatitude: 18°03.062N; verbatimLongitude: 76°05.356E; verbatimCoordinateSystem: degrees minutes; geodeticDatum: WGS84; **Event:** month: October-February and May-June; fieldNumber: *R.D. Gore-* 13189; fieldNotes: Erect or prostrate herbs; **Record Level:** institutionCode: Wachland College of Arts & Science, Solapur (WCAS).

#### Butea monospermavar.monosperma

(Lam.) Taub. 1894

##### Materials

- **Type status:** Other material. **Location:** continent: Asia; country: India; countryCode: IN; stateProvince: Maharashtra; municipality: Latur; locality: Chakur; verbatimLatitude: 18°31.874N; verbatimLongitude: 76°54.516E; verbatimCoordinateSystem: degrees minutes; geodeticDatum: WGS84; **Event:** month: March-June; fieldNumber: *RDG-* 046; fieldNotes: Trees; **Record Level:** institutionCode: Wachland College of Arts & Science, Solapur (WCAS).
- **Type status:** Other material. **Location:** continent: Asia; country: India; countryCode: IN; stateProvince: Maharashtra; municipality: Tuljapur; locality: Naldurg; verbatimLatitude: 17°47.741N; verbatimLongitude: 76°17.807E; verbatimCoordinateSystem: degrees minutes; geodeticDatum: WGS84; **Event:** month: March-June; fieldNumber: *R.D. Gore-* 13062; fieldNotes: Trees; **Record Level:** institutionCode: Wachland College of Arts & Science, Solapur (WCAS).

#### Cajanus cajan

(L.) Mill. 1900

##### Materials

- **Type status:** Other material. **Location:** continent: Asia; country: India; countryCode: IN; stateProvince: Maharashtra; municipality: Tuljapur; locality: Chiwri; verbatimLatitude: 17°52.201N; verbatimLongitude: 76°12.725E; verbatimCoordinateSystem: degrees minutes; geodeticDatum: WGS84; **Event:** month: September-February; fieldNumber: *R.D. Gore-* 13163; fieldNotes: Shrubs; **Record Level:** institutionCode: Wachland College of Arts & Science, Solapur (WCAS).

#### Cajanus platycarpus

(Benth.) van der Maesen, 1986

##### Materials

- **Type status:** Other material. **Location:** continent: Asia; country: India; countryCode: IN; stateProvince: Maharashtra; municipality: Osmanabad; locality: Ramling; verbatimLatitude: 18°17.408N; verbatimLongitude: 75°57.347E; verbatimCoordinateSystem: degrees minutes; geodeticDatum: WGS84; **Event:** month: September-December; fieldNumber: *RDG-* 745; fieldNotes: Trailing/twining herbs; **Record Level:** institutionCode: Wachland College of Arts & Science, Solapur (WCAS).

#### Cajanus scarabaeoides

(L.) Thouars, 1817

##### Materials

- **Type status:** Other material. **Location:** continent: Asia; country: India; countryCode: IN; stateProvince: Maharashtra; municipality: Ambajogai; locality: Near Talni; verbatimLatitude: 18°44.377N; verbatimLongitude: 76°31.028E; verbatimCoordinateSystem: degrees minutes; geodeticDatum: WGS84; **Event:** month: September-December; fieldNumber: *RDG-* 021; fieldNotes: Trailing/twining herbs; **Record Level:** institutionCode: Wachland College of Arts & Science, Solapur (WCAS).

#### Canavalia cathartica

Thouars, 1991

##### Materials

- **Type status:** Other material. **Location:** continent: Asia; country: India; countryCode: IN; stateProvince: Maharashtra; municipality: Patoda; locality: Near Khandala; verbatimLatitude: 18°50.423N; verbatimLongitude: 75°41.969E; verbatimCoordinateSystem: degrees minutes; geodeticDatum: WGS84; **Event:** month: September-December; fieldNumber: *RDG-* 1018; fieldNotes: Woody climbers; **Record Level:** institutionCode: Wachland College of Arts & Science, Solapur (WCAS).

#### Canavalia gladiata

(Jacq.) DC. 1825

##### Materials

- **Type status:** Other material. **Location:** continent: Asia; country: India; countryCode: IN; stateProvince: Maharashtra; municipality: Barshi; locality: Ukkadgaon; verbatimLatitude: 18°20.458N; verbatimLongitude: 75°53.747E; verbatimCoordinateSystem: degrees minutes; geodeticDatum: WGS84; **Event:** month: August-December; fieldNumber: *KUG-* 430; fieldNotes: Woody climbers; **Record Level:** institutionCode: Wachland College of Arts & Science, Solapur (WCAS).

#### Cicer arietinum

L. 1753

##### Materials

- **Type status:** Other material. **Location:** continent: Asia; country: India; countryCode: IN; stateProvince: Maharashtra; municipality: Ambajogai; locality: Near Talni; verbatimLatitude: 18°44.277N; verbatimLongitude: 76°30.874E; verbatimCoordinateSystem: degrees minutes; geodeticDatum: WGS84; **Event:** month: November-January; fieldNumber: *RDG-* 022; fieldNotes: Erect herbs; **Record Level:** institutionCode: Wachland College of Arts & Science, Solapur (WCAS).
- **Type status:** Other material. **Location:** continent: Asia; country: India; countryCode: IN; stateProvince: Maharashtra; municipality: Tuljapur; locality: Apsinga; verbatimLatitude: 18°04.951N; verbatimLongitude: 76°01.676E; verbatimCoordinateSystem: degrees minutes; geodeticDatum: WGS84; **Event:** month: November-January; fieldNumber: *R.D. Gore-* 13114; fieldNotes: Erect herbs; **Record Level:** institutionCode: Wachland College of Arts & Science, Solapur (WCAS).

#### Clitoria ternateavar.pilosula

Wall. ex Baker, 1876

##### Materials

- **Type status:** Other material. **Location:** continent: Asia; country: India; countryCode: IN; stateProvince: Maharashtra; municipality: Tuljapur; locality: Jalkot; verbatimLatitude: 17°49.521N; verbatimLongitude: 76°20.436E; verbatimCoordinateSystem: degrees minutes; geodeticDatum: WGS84; **Event:** month: July-October; fieldNumber: *RDG-* 355; fieldNotes: Trailing/twining herbs; **Record Level:** institutionCode: Wachland College of Arts & Science, Solapur (WCAS).

#### Clitoria ternateavar.ternatea

L. 1753

##### Materials

- **Type status:** Other material. **Location:** continent: Asia; country: India; countryCode: IN; stateProvince: Maharashtra; municipality: Tuljapur; locality: Apsinga; verbatimLatitude: 18°04. 661N; verbatimLongitude: 76°01.855E; verbatimCoordinateSystem: degrees minutes; geodeticDatum: WGS84; **Event:** month: August-December; fieldNumber: **R. D.* Gore-* 13126; fieldNotes: Trailing/twining herbs; **Record Level:** institutionCode: Wachland College of Arts & Science, Solapur (WCAS).

#### Crotalaria albida

Heyne ex Roth, 1821

##### Materials

- **Type status:** Other material. **Location:** continent: Asia; country: India; countryCode: IN; stateProvince: Maharashtra; municipality: Beed; locality: Bhayala; verbatimLatitude: 18°51.525N; verbatimLongitude: 75°37.058E; verbatimCoordinateSystem: degrees minutes; geodeticDatum: WGS84; **Event:** month: September-January; fieldNumber: *RDG-* 1289; fieldNotes: Erect herbs; **Record Level:** institutionCode: Wachland College of Arts & Science, Solapur (WCAS).

#### Crotalaria berteroana

DC. 1825

##### Materials

- **Type status:** Other material. **Location:** continent: Asia; country: India; countryCode: IN; stateProvince: Maharashtra; county: Marathwada; municipality: Osmanabad; **Event:** month: November-February; fieldNumber: *Almeida-* s.n.; fieldNotes: Shrubs; **Record Level:** institutionCode: Blatter Herbaium, St. Xavier’s College, Mumbai (BLAT).

#### Crotalaria decasperma

Naik, 1966

##### Materials

- **Type status:** Other material. **Location:** continent: Asia; country: India; countryCode: IN; stateProvince: Maharashtra; municipality: Osmanabad; locality: Ramling sanctuary; verbatimLatitude: 18°17.584N; verbatimLongitude: 75°57.059E; verbatimCoordinateSystem: degrees minutes; geodeticDatum: WGS84; **Event:** month: September-November; fieldNumber: *RDG-* 218; fieldNotes: Erect herbs; **Record Level:** institutionCode: Wachland College of Arts & Science, Solapur (WCAS).

#### Crotalaria ferruginea

Grah. ex Benth. 1843

##### Materials

- **Type status:** Other material. **Location:** continent: Asia; country: India; countryCode: IN; stateProvince: Maharashtra; municipality: Nilanga; locality: Tambala; verbatimLatitude: 17°56.534N; verbatimLongitude: 76°53.307E; verbatimCoordinateSystem: degrees minutes; geodeticDatum: WGS84; **Event:** month: November-February; fieldNumber: *RDG-* 1057; fieldNotes: Erect herbs; **Record Level:** institutionCode: Wachland College of Arts & Science, Solapur (WCAS).

#### Crotalaria filipes

Benth. 1843

##### Materials

- **Type status:** Other material. **Location:** continent: Asia; country: India; countryCode: IN; stateProvince: Maharashtra; municipality: Ausa; locality: Killari; verbatimLatitude: 18°03.570N; verbatimLongitude: 76°35.565E; verbatimCoordinateSystem: degrees minutes; geodeticDatum: WGS84; **Event:** month: August-October; fieldNumber: *RDG-* 243; fieldNotes: Erect herbs; **Record Level:** institutionCode: Wachland College of Arts & Science, Solapur (WCAS).

#### Crotalaria hebecarpa

(DC.) Rudd, 1983

##### Materials

- **Type status:** Other material. **Location:** continent: Asia; country: India; countryCode: IN; stateProvince: Maharashtra; municipality: Ambajogai; locality: Near Pus; verbatimLatitude: 18°44.471N; verbatimLongitude: 76°29.617E; verbatimCoordinateSystem: degrees minutes; geodeticDatum: WGS84; **Event:** month: July-December; fieldNumber: *RDG-* 018; fieldNotes: Erect herbs; **Record Level:** institutionCode: Wachland College of Arts & Science, Solapur (WCAS).

#### Crotalaria hirta

Willd. 1803

##### Materials

- **Type status:** Other material. **Location:** continent: Asia; country: India; countryCode: IN; stateProvince: Maharashtra; municipality: Osmanabad; locality: Ramling sanctuary; verbatimLatitude: 18°17.491N; verbatimLongitude: 75°57.145E; verbatimCoordinateSystem: degrees minutes; geodeticDatum: WGS84; **Event:** month: July-December; fieldNumber: *RDG-* 949; fieldNotes: Erect herbs; **Record Level:** institutionCode: Wachland College of Arts & Science, Solapur (WCAS).

#### Crotalaria juncea

L. 1753

##### Materials

- **Type status:** Other material. **Location:** continent: Asia; country: India; countryCode: IN; stateProvince: Maharashtra; municipality: Latur; locality: Nagzari; verbatimLatitude: 18°28.025N; verbatimLongitude: 76°30.667E; verbatimCoordinateSystem: degrees minutes; geodeticDatum: WGS84; **Event:** month: July-December; fieldNumber: *RDG-* 482; fieldNotes: Erect herbs; **Record Level:** institutionCode: Wachland College of Arts & Science, Solapur (WCAS).

#### Crotalaria leptostachya

Benth. 1843

##### Materials

- **Type status:** Other material. **Location:** continent: Asia; country: India; countryCode: IN; stateProvince: Maharashtra; municipality: Osmanabad; locality: Near Alni; verbatimLatitude: 18°16.863N; verbatimLongitude: 76°00.298E; verbatimCoordinateSystem: degrees minutes; geodeticDatum: WGS84; **Event:** month: July-December; fieldNumber: *RDG-* 307; fieldNotes: Erect herbs; **Record Level:** institutionCode: Wachland College of Arts & Science, Solapur (WCAS).

#### Crotalaria linifolia

L. f. 1781

##### Materials

- **Type status:** Other material. **Location:** continent: Asia; country: India; countryCode: IN; stateProvince: Maharashtra; municipality: Washi (Osmanabad); locality: Dindori; verbatimLatitude: 18°26.741N; verbatimLongitude: 75°46.191E; verbatimCoordinateSystem: degrees minutes; geodeticDatum: WGS84; **Event:** month: August-November; fieldNumber: *RDG-* 1275; fieldNotes: Shrubs; **Record Level:** institutionCode: Wachland College of Arts & Science, Solapur (WCAS).

#### Crotalaria medicaginea

Lam. 1786

##### Materials

- **Type status:** Other material. **Location:** continent: Asia; country: India; countryCode: IN; stateProvince: Maharashtra; municipality: Dharur (Beed); locality: Jiwachiwadi; verbatimLatitude: 18°50.570N; verbatimLongitude: 76°00.963E; verbatimCoordinateSystem: degrees minutes; geodeticDatum: WGS84; **Event:** month: August-October; fieldNumber: *RDG-* 1278; fieldNotes: Erect herbs; **Record Level:** institutionCode: Wachland College of Arts & Science, Solapur (WCAS).

#### Crotalaria montana

Heyne ex Roth, 1821

##### Materials

- **Type status:** Other material. **Location:** continent: Asia; country: India; countryCode: IN; stateProvince: Maharashtra; municipality: Barshi; locality: Chumb; verbatimLatitude: 18°24.714N; verbatimLongitude: 75°45.386E; verbatimCoordinateSystem: degrees minutes; geodeticDatum: WGS84; **Event:** month: August-December; fieldNumber: *RDG-* 1197; fieldNotes: Erect herbs; **Record Level:** institutionCode: Wachland College of Arts & Science, Solapur (WCAS).

#### Crotalaria mysorensis

Roth, 1821

##### Materials

- **Type status:** Other material. **Location:** continent: Asia; country: India; countryCode: IN; stateProvince: Maharashtra; municipality: Ambajogai; locality: Mukundraj; verbatimLatitude: 18°45.567N; verbatimLongitude: 76°22.043E; verbatimCoordinateSystem: degrees minutes; geodeticDatum: WGS84; **Event:** month: August-January; fieldNumber: *RDG-* 385; fieldNotes: Erect herbs; **Record Level:** institutionCode: Wachland College of Arts & Science, Solapur (WCAS).

#### Crotalaria notonii

Wight & Arn. 1834

##### Materials

- **Type status:** Other material. **Location:** continent: Asia; country: India; countryCode: IN; stateProvince: Maharashtra; municipality: Patoda; locality: Wadzari; verbatimLatitude: 18°54.413N; verbatimLongitude: 75°32.010E; verbatimCoordinateSystem: degrees minutes; geodeticDatum: WGS84; **Event:** month: September-December; fieldNumber: *RDG-* 720; fieldNotes: Erect herbs; **Record Level:** institutionCode: Wachland College of Arts & Science, Solapur (WCAS).

#### Crotalaria orixensis

Willd. 1803

##### Materials

- **Type status:** Other material. **Location:** continent: Asia; country: India; countryCode: IN; stateProvince: Maharashtra; municipality: Osmanabad; locality: Ramling sanctuary; verbatimLatitude: 18°17.799N; verbatimLongitude: 75°56.720E; verbatimCoordinateSystem: degrees minutes; geodeticDatum: WGS84; **Event:** month: August-December; fieldNumber: *RDG-* 007; fieldNotes: Trailing/twining herbs; **Record Level:** institutionCode: Wachland College of Arts & Science, Solapur (WCAS).

#### Crotalaria pallida

Ait. 1789

##### Materials

- **Type status:** Other material. **Location:** continent: Asia; country: India; countryCode: IN; stateProvince: Maharashtra; municipality: Barshi (Solapur); locality: Barshi town; verbatimLatitude: 18°15.257N; verbatimLongitude: 75°42.443E; verbatimCoordinateSystem: degrees minutes; geodeticDatum: WGS84; **Event:** month: August-January; fieldNumber: *RDG-* 676; fieldNotes: Erect herbs; **Record Level:** institutionCode: Wachland College of Arts & Science, Solapur (WCAS).

#### Crotalaria prostrata

Rottl. 1809

##### Materials

- **Type status:** Other material. **Location:** continent: Asia; country: India; countryCode: IN; stateProvince: Maharashtra; municipality: Ambajogai; locality: Near Ghatnandur; verbatimLatitude: 18°43.878N; verbatimLongitude: 76°32.367E; verbatimCoordinateSystem: degrees minutes; geodeticDatum: WGS84; **Event:** month: September-December; fieldNumber: *RDG-* 015; fieldNotes: Erect herbs; **Record Level:** institutionCode: Wachland College of Arts & Science, Solapur (WCAS).

#### Crotalaria pusilla

Heyne ex DC. 1825

##### Materials

- **Type status:** Other material. **Location:** continent: Asia; country: India; countryCode: IN; stateProvince: Maharashtra; municipality: Beed; locality: Near Yelamb-ghat; verbatimLatitude: 18°47.282N; verbatimLongitude: 75°48.548E; verbatimCoordinateSystem: degrees minutes; geodeticDatum: WGS84; **Event:** month: August-November; fieldNumber: *RDG-* 386; fieldNotes: Erect herbs; **Record Level:** institutionCode: ​Wachland College of Arts & Science, Solapur (WCAS).

#### Crotalaria ramosissima

Roxb. 1832

##### Materials

- **Type status:** Other material. **Location:** continent: Asia; country: India; countryCode: IN; stateProvince: Maharashtra; municipality: Lohara; locality: Near Wadgaon; verbatimLatitude: 17°55.868N; verbatimLongitude: 76°20.933E; verbatimCoordinateSystem: degrees minutes; geodeticDatum: WGS84; **Event:** month: September-February; fieldNumber: *RDG-* 483; fieldNotes: Shrubs; **Record Level:** institutionCode: Walchand College of Arts & Science, Solapur (WCAS).

#### Crotalaria retusa

L. 1753

##### Materials

- **Type status:** Other material. **Location:** continent: Asia; country: India; countryCode: IN; stateProvince: Maharashtra; municipality: Ambajogai; locality: Murkatwadi; verbatimLatitude: 18°44.280N; verbatimLongitude: 76°35.551E; verbatimCoordinateSystem: degrees minutes; geodeticDatum: WGS84; **Event:** month: October-January; fieldNumber: *RDG*- 027; fieldNotes: Shrubs; **Record Level:** institutionCode: Wachland College of Arts & Science, Solapur (WCAS).

#### Crotalaria spectabilis

Roth, 1821

##### Materials

- **Type status:** Other material. **Location:** continent: Asia; country: India; countryCode: IN; stateProvince: Maharashtra; municipality: Ambajogai; locality: Mukundraj; verbatimLatitude: 18°45.567N; verbatimLongitude: 76°22.043E; verbatimCoordinateSystem: degrees minutes; geodeticDatum: WGS84; **Event:** month: October-January; fieldNumber: *RDG*- 735; fieldNotes: Shrubs; **Record Level:** institutionCode: Wachland College of Arts & Science, Solapur (WCAS).

#### Crotalaria verrucosa

L. 1753

##### Materials

- **Type status:** Other material. **Location:** continent: Asia; country: India; countryCode: IN; stateProvince: Maharashtra; municipality: Ambajogai; locality: Near Talni; verbatimLatitude: 18°44.244N; verbatimLongitude: 76°30.519E; verbatimCoordinateSystem: degrees minutes; geodeticDatum: WGS84; **Event:** month: August-November; fieldNumber: *RDG*- 026; fieldNotes: Erect herbs; **Record Level:** institutionCode: Wachland College of Arts & Science, Solapur (WCAS).

#### Crotalaria vestita

Baker, 1876

##### Materials

- **Type status:** Other material. **Location:** continent: Asia; country: India; countryCode: IN; stateProvince: Maharashtra; municipality: Osmanabad; locality: Gad-devdari; verbatimLatitude: 18°15.772N; verbatimLongitude: 75°59.455E; verbatimCoordinateSystem: degrees minutes; geodeticDatum: WGS84; **Event:** month: September-March; fieldNumber: *RDG*- 1015; fieldNotes: Erect herbs; **Record Level:** institutionCode: Wachland College of Arts & Science, Solapur (WCAS).

#### Cullen corylifolia

(L.) Medik. 1787

##### Materials

- **Type status:** Other material. **Location:** continent: Asia; country: India; countryCode: IN; stateProvince: Maharashtra; municipality: Chakur; locality: Gharni-dam (Naleagon); verbatimLatitude: 18°22.545N; verbatimLongitude: 76°50.148E; verbatimCoordinateSystem: degrees minutes; geodeticDatum: WGS84; **Event:** month: August-December; fieldNumber: *RDG*- 531; fieldNotes: Erect herbs; **Record Level:** institutionCode: Wachland College of Arts & Science, Solapur (WCAS).
- **Type status:** Other material. **Location:** continent: Asia; country: India; countryCode: IN; stateProvince: Maharashtra; municipality: Tuljapur; locality: Apsinga; verbatimLatitude: 18°03.327N; verbatimLongitude: 76°02.454E; verbatimCoordinateSystem: degrees minutes; geodeticDatum: WGS84; **Event:** month: August-December; fieldNumber: *R.D. Gore-* 13101; fieldNotes: Erect herbs; **Record Level:** institutionCode: Wachland College of Arts & Science, Solapur (WCAS).

#### Cyamopsis tetragonoloba

(L.) Taub. 1894

##### Materials

- **Type status:** Other material. **Location:** continent: Asia; country: India; countryCode: IN; stateProvince: Maharashtra; municipality: Ambajogai; locality: Near Pus; verbatimLatitude: 18°44.206N; verbatimLongitude: 76°29.988E; verbatimCoordinateSystem: degrees minutes; geodeticDatum: WGS84; **Event:** month: January-December; fieldNumber: *RDG*- 019; fieldNotes: Erect herbs; **Record Level:** institutionCode: Wachland College of Arts & Science, Solapur (WCAS).
- **Type status:** Other material. **Location:** continent: Asia; country: India; countryCode: IN; stateProvince: Maharashtra; municipality: Tuljapur; locality: Apsinga; verbatimLatitude: 18°04.951N; verbatimLongitude: 76°01.676E; verbatimCoordinateSystem: degrees minutes; geodeticDatum: WGS84; **Event:** month: January-December; fieldNumber: *R.D. Gore-* 13030; fieldNotes: Erect herbs; **Record Level:** institutionCode: Wachland College of Arts & Science, Solapur (WCAS).

#### Dalbergia lanceolaria lanceolaria

L. f. 1781

##### Materials

- **Type status:** Other material. **Location:** continent: Asia; country: India; countryCode: IN; stateProvince: Maharashtra; municipality: Beed; locality: Dharur; verbatimLatitude: 18°49.845N; verbatimLongitude: 76°06.961E; verbatimCoordinateSystem: degrees minutes; geodeticDatum: WGS84; **Event:** month: March-June; fieldNumber: *RDG*- 1314; fieldNotes: Trees; **Record Level:** institutionCode: Wachland College of Arts & Science, Solapur (WCAS).

#### Dalbergia lanceolaria paniculata

(Roxb.) Thoth. 1985

##### Materials

- **Type status:** Other material. **Location:** continent: Asia; country: India; countryCode: IN; stateProvince: Maharashtra; municipality: Renapur; locality: Karepur; verbatimLatitude: 18°35.471N; verbatimLongitude: 76°41.795E; verbatimCoordinateSystem: degrees minutes; geodeticDatum: WGS84; **Event:** month: March-July; fieldNumber: *RDG*- 615; fieldNotes: Trees; **Record Level:** institutionCode: Wachland College of Arts & Science, Solapur (WCAS).

#### Dalbergia latifolia

Roxb. 1799

##### Materials

- **Type status:** Other material. **Location:** continent: Asia; country: India; countryCode: IND; stateProvince: Maharashtra; municipality: Kalamb; locality: Ranjani; verbatimLatitude: 18°32.116N; verbatimLongitude: 76°14.515E; verbatimCoordinateSystem: degrees minutes; geodeticDatum: WGS84; **Event:** month: July-October; fieldNumber: *RDG*- 129; fieldNotes: Trees; **Record Level:** institutionCode: Wachland College of Arts & Science, Solapur (WCAS).

#### Dalbergia melanoxylon

Guill. & Perr. 1832

##### Materials

- **Type status:** Other material. **Location:** continent: Asia; country: India; countryCode: IN; stateProvince: Maharashtra; municipality: Chakur; locality: Wagholi; verbatimLatitude: 18°33.873N; verbatimLongitude: 76°48.690E; verbatimCoordinateSystem: degrees minutes; geodeticDatum: WGS84; **Event:** month: April-June; fieldNumber: *RDG*- 375; fieldNotes: Trees; **Record Level:** institutionCode: Wachland College of Arts & Science, Solapur (WCAS).

#### Dalbergia sissoo

Roxb. ex DC. 1825

##### Materials

- **Type status:** Other material. **Location:** continent: Asia; country: India; countryCode: IN; stateProvince: Maharashtra; municipality: Tuljapur; locality: Papnas; verbatimLatitude: 18°00.164N; verbatimLongitude: 76°04.154E; verbatimCoordinateSystem: degrees minutes; geodeticDatum: WGS84; **Event:** month: March-December; fieldNumber: *RDG*- 648; fieldNotes: Trees; **Record Level:** institutionCode: Wachland College of Arts & Science, Solapur (WCAS).
- **Type status:** Other material. **Location:** continent: Asia; country: India; countryCode: IN; stateProvince: Maharashtra; municipality: Tuljapur; locality: Apsinga road; verbatimLatitude: 18°04.142N; verbatimLongitude: 76°04.220E; verbatimCoordinateSystem: degrees minutes; geodeticDatum: WGS84; **Event:** month: March-December; fieldNumber: *R.D. Gore-* 13141; fieldNotes: Trees; **Record Level:** institutionCode: Wachland College of Arts & Science, Solapur (WCAS).

#### Desmodium alysicarpoides

van Meeuwen, 1962

##### Materials

- **Type status:** Other material. **Location:** continent: Asia; country: India; countryCode: IN; stateProvince: Maharashtra; municipality: Osmanabad; locality: Ramling sanctury; verbatimLatitude: 18°17.648N; verbatimLongitude: 75°56.928E; verbatimCoordinateSystem: degrees minutes; geodeticDatum: WGS84; **Event:** month: September-December; fieldNumber: *RDG*- 743; fieldNotes: Erect herbs; **Record Level:** institutionCode: Wachland College of Arts & Science, Solapur (WCAS).

#### Desmodium dichotomum

(Willd.) DC. 1825

##### Materials

- **Type status:** Other material. **Location:** continent: Asia; country: India; countryCode: IN; stateProvince: Maharashtra; municipality: Beed; locality: Pachangri; verbatimLatitude: 18°43.013N; verbatimLongitude: 75°35.435E; verbatimCoordinateSystem: degrees minutes; geodeticDatum: WGS84; **Event:** month: September-January; fieldNumber: *RDG*- 1242; fieldNotes: Erect herbs; **Record Level:** institutionCode: Wachland College of Arts & Science, Solapur (WCAS).

#### Desmodium gangeticum

(L.) DC. 1825

##### Materials

- **Type status:** Other material. **Location:** continent: Asia; country: India; countryCode: IN; stateProvince: Maharashtra; municipality: Osmanabad; locality: Bhanasgaon; verbatimLatitude: 18°17.094N; verbatimLongitude: 75°56.724E; verbatimCoordinateSystem: degrees minutes; geodeticDatum: WGS84; **Event:** month: September-January; fieldNumber: *RDG*- 010; fieldNotes: Shrubs; **Record Level:** institutionCode: Wachland College of Arts & Science, Solapur (WCAS).

#### Desmodium heterocarpon

(L.) DC. 1825

##### Materials

- **Type status:** Other material. **Location:** continent: Asia; country: India; countryCode: IN; stateProvince: Maharashtra; municipality: Solapur; locality: Solapur district; **Event:** month: August-September; fieldNumber: *Kothari*- s.n.; fieldNotes: Shrubs; **Record Level:** institutionCode: Botanical Survey of India (BSI).

#### Desmodium laxiflorum

DC. 1825

##### Materials

- **Type status:** Other material. **Location:** continent: Asia; country: India; countryCode: IN; stateProvince: Maharashtra; municipality: Beed; locality: Bhayala; verbatimLatitude: 18°51.753N; verbatimLongitude: 75°36.987E; verbatimCoordinateSystem: degrees minutes; geodeticDatum: WGS84; **Event:** month: August-December; fieldNumber: *RDG*- 1290; fieldNotes: Shrubs; **Record Level:** institutionCode: Wachland College of Arts & Science, Solapur (WCAS).

#### Desmodium oojeinense

(Roxb.) Ohashi, 1973

##### Materials

- **Type status:** Other material. **Location:** continent: Asia; country: India; countryCode: IN; stateProvince: Maharashtra; municipality: Patoda; locality: Gangewadi; verbatimLatitude: 18°54.025N; verbatimLongitude: 75°14.831E; verbatimCoordinateSystem: degrees minutes; geodeticDatum: WGS84; **Event:** month: March-May; fieldNumber: *RDG*- 682; fieldNotes: Trees; **Record Level:** institutionCode: Wachland College of Arts & Science, Solapur (WCAS).

#### Desmodium triflorum

(L.) DC. 1825

##### Materials

- **Type status:** Other material. **Location:** continent: Asia; country: India; countryCode: IN; stateProvince: Maharashtra; municipality: Barshi; locality: Wakadi; verbatimLatitude: 18°16.004N; verbatimLongitude: 75°34.095E; verbatimCoordinateSystem: degrees minutes; geodeticDatum: WGS84; **Event:** month: September-March; fieldNumber: *RDG*- 045; fieldNotes: Trailing/twining herbs; **Record Level:** institutionCode: Wachland College of Arts & Science, Solapur (WCAS).

#### Desmodium velutinum

(Willd.) DC. 1825

##### Materials

- **Type status:** Other material. **Location:** continent: Asia; country: India; countryCode: IN; stateProvince: Maharashtra; municipality: Osmanabad; locality: Ghatangri; verbatimLatitude: 18°13.292N; verbatimLongitude: 76°01.252E; verbatimCoordinateSystem: degrees minutes; geodeticDatum: WGS84; **Event:** month: September-January; fieldNumber: *RDG*- 799; fieldNotes: Shrubs; **Record Level:** institutionCode: Wachland College of Arts & Science, Solapur (WCAS).

#### Dunbaria glandulosa

(Dalz.) Prain, 1897

##### Materials

- **Type status:** Other material. **Location:** continent: Asia; country: India; countryCode: IN; stateProvince: Maharashtra; municipality: Chakur; locality: Wagholi; verbatimLatitude: 18°34.163N; verbatimLongitude: 76°49.546E; verbatimCoordinateSystem: degrees minutes; geodeticDatum: WGS84; **Event:** month: August-October; fieldNumber: *RDG*- 1268; fieldNotes: Woody climbers; **Record Level:** institutionCode: Wachland College of Arts & Science, Solapur (WCAS).

#### Erythrina suberosa

Roxb. 1832

##### Materials

- **Type status:** Other material. **Location:** continent: Asia; country: India; countryCode: IND; stateProvince: Maharashtra; municipality: Tuljapur; locality: Apsinga; verbatimLatitude: 18°04.845N; verbatimLongitude: 76°02.001E; verbatimCoordinateSystem: degrees minutes; geodeticDatum: WGS84; **Event:** month: February-May; fieldNumber: *RDG*- 043; fieldNotes: Trees; **Record Level:** institutionCode: Wachland College of Arts & Science, Solapur (WCAS).

#### Erythrina variegata

L. 1754

##### Materials

- **Type status:** Other material. **Location:** continent: Asia; country: India; countryCode: IN; stateProvince: Maharashtra; municipality: Beed; locality: Yelamb-ghat; verbatimLatitude: 18°47.469N; verbatimLongitude: 75°49.470E; verbatimCoordinateSystem: degrees minutes; geodeticDatum: WGS84; **Event:** month: February-June; fieldNumber: *RDG*- 923; fieldNotes: Trees; **Record Level:** institutionCode: Wachland College of Arts & Science, Solapur (WCAS).

#### Flemingia strobilifera

(L.) R. Br. 1812

##### Materials

- **Type status:** Other material. **Location:** continent: Asia; country: India; countryCode: IND; stateProvince: Maharashtra; municipality: Pathardi; locality: Manewadi; verbatimLatitude: 19°02.440N; verbatimLongitude: 75°16.957E; verbatimCoordinateSystem: degrees minutes; geodeticDatum: WGS84; **Event:** month: August-September; fieldNumber: *RDG*- 617; fieldNotes: Shrubs; **Record Level:** institutionCode: Wachland College of Arts & Science, Solapur (WCAS).

#### Gliricidia sepium

(Jacq.) Kunth ex Walp. 1842

##### Materials

- **Type status:** Other material. **Location:** continent: Asia; country: India; countryCode: IN; stateProvince: Maharashtra; municipality: Udgir; locality: Dongraj; verbatimLatitude: 18°29.675N; verbatimLongitude: 77°01.694E; verbatimCoordinateSystem: degrees minutes; geodeticDatum: WGS84; **Event:** month: February-June; fieldNumber: *RDG*- 550; fieldNotes: Trees; **Record Level:** institutionCode: Wachland College of Arts & Science, Solapur (WCAS).

#### Glycine max

(L.) Merr. 1917

##### Materials

- **Type status:** Other material. **Location:** continent: Asia; country: India; countryCode: IN; stateProvince: Maharashtra; municipality: Ambajogai; locality: Mukundraj; verbatimLatitude: 18°45.134N; verbatimLongitude: 76°21.867E; verbatimCoordinateSystem: degrees minutes; geodeticDatum: WGS84; **Event:** month: August-December; fieldNumber: *RDG*- 736; fieldNotes: Erect herbs; **Record Level:** institutionCode: Wachland College of Arts & Science, Solapur (WCAS).

#### Glycyrrhiza glabra

L. 1753

##### Materials

- **Type status:** Other material. **Location:** continent: Asia; country: India; countryCode: IN; stateProvince: Maharashtra; municipality: Osmanabad; locality: Government Agriculture College, Kini; **Event:** month: June-October; fieldNumber: *RDG*- 785; fieldNotes: Erect herbs; **Record Level:** institutionCode: Wachland College of Arts & Science, Solapur (WCAS).

#### Indigofera astragalina

DC. 1825

##### Materials

- **Type status:** Other material. **Location:** continent: Asia; country: India; countryCode: IN; stateProvince: Maharashtra; municipality: Osmanabad; locality: Ramling; verbatimLatitude: 18°17.447N; verbatimLongitude: 75°57.178E; verbatimCoordinateSystem: degrees minutes; geodeticDatum: WGS84; **Event:** month: August-November; fieldNumber: *RDG*- 763; fieldNotes: Erect herbs; **Record Level:** institutionCode: Wachland College of Arts & Science, Solapur (WCAS).

#### Indigofera cassioides

Rottl. ex DC. 1825

##### Materials

- **Type status:** Other material. **Location:** continent: Asia; country: India; countryCode: IN; stateProvince: Maharashtra; municipality: Beed; locality: Naigaon (PT); verbatimLatitude: 18°53.501N; verbatimLongitude: 75°35.364E; verbatimCoordinateSystem: degrees minutes; geodeticDatum: WGS84; **Event:** month: July-December; fieldNumber: *RDG*- 967; fieldNotes: Shrubs; **Record Level:** institutionCode: Wachland College of Arts & Science, Solapur (WCAS).

#### Indigofera coerulea

Roxb. 1832

##### Materials

- **Type status:** Other material. **Location:** continent: Asia; country: India; countryCode: IN; stateProvince: Maharashtra; municipality: Ambajogai; locality: Ghatnandur; verbatimLatitude: 18°43.875N; verbatimLongitude: 76°33.351E; verbatimCoordinateSystem: degrees minutes; geodeticDatum: WGS84; **Event:** month: September- December; fieldNumber: *RDG*- 013; fieldNotes: Shrubs; **Record Level:** institutionCode: Wachland College of Arts & Science, Solapur (WCAS).

#### Indigofera cordifolia

Heyne ex Roth. 1821

##### Materials

- **Type status:** Other material. **Location:** continent: Asia; country: India; countryCode: IN; stateProvince: Maharashtra; municipality: Naldurg; locality: Jalkot; verbatimLatitude: 17°49.054N; verbatimLongitude: 76°20.298E; verbatimCoordinateSystem: degrees minutes; geodeticDatum: WGS84; **Event:** month: July-November; fieldNumber: *RDG*- 362; fieldNotes: Erect herbs; **Record Level:** institutionCode: Wachland College of Arts & Science, Solapur (WCAS).

#### Indigofera deccanensis

Sanjappa, 1983

##### Materials

- **Type status:** Other material. **Location:** continent: Asia; country: India; countryCode: IN; stateProvince: Maharashtra; municipality: Patoda (Beed); locality: Sautada; verbatimLatitude: 18°47.927N; verbatimLongitude: 75°21.069E; verbatimCoordinateSystem: degrees minutes; geodeticDatum: WGS84; **Event:** month: August-December; fieldNumber: *RDG*- 999; fieldNotes: Shrubs; **Record Level:** institutionCode: Wachland College of Arts & Science, Solapur (WCAS).

#### Indigofera glandulosavar.glandulosa

Wendl. 1748

##### Materials

- **Type status:** Other material. **Location:** continent: Asia; country: India; countryCode: IN; stateProvince: Maharashtra; municipality: Ambajogai; locality: Near Talni; verbatimLatitude: 18°44.377N; verbatimLongitude: 76°30.592E; verbatimCoordinateSystem: degrees minutes; geodeticDatum: WGS84; **Event:** month: August-January; fieldNumber: *RDG*- 020; fieldNotes: Erect herbs; **Record Level:** institutionCode: Wachland College of Arts & Science, Solapur (WCAS).

#### Indigofera glandulosavar.sykesii

Griff. ex Baker, 1876

##### Materials

- **Type status:** Other material. **Location:** continent: Asia; country: India; countryCode: IN; stateProvince: Maharashtra; municipality: Osmanabad; locality: Ramling; verbatimLatitude: 18°17.464N; verbatimLongitude: 75°57.168E; verbatimCoordinateSystem: degrees minutes; geodeticDatum: WGS84; **Event:** month: August-November; fieldNumber: *RDG*- 298; fieldNotes: Erect herb; **Record Level:** institutionCode: Wachland College of Arts & Science, Solapur (WCAS).

#### Indigofera linifolia

(L. f) Retz. 1786

##### Materials

- **Type status:** Other material. **Location:** continent: Asia; country: India; countryCode: IN; stateProvince: Maharashtra; municipality: Osmanabad; locality: Ramling; verbatimLatitude: 18°17.918N; verbatimLongitude: 75°57.126E; verbatimCoordinateSystem: degrees minutes; geodeticDatum: WGS84; **Event:** month: August-January; fieldNumber: *RDG*- 002; fieldNotes: Erect herbs; **Record Level:** institutionCode: Wachland College of Arts & Science, Solapur (WCAS).

#### Indigofera linnaei

Ali, 1958

##### Materials

- **Type status:** Other material. **Location:** continent: Asia; country: India; countryCode: IN; stateProvince: Maharashtra; municipality: Ambajogai; locality: Talni; verbatimLatitude: 18°44.131N; verbatimLongitude: 76°30.630E; verbatimCoordinateSystem: degrees minutes; geodeticDatum: WGS84; **Event:** month: June-December; fieldNumber: *RDG*- 011; fieldNotes: Trailing/twining herbs; **Record Level:** institutionCode: Wachland College of Arts & Science, Solapur (WCAS).

#### Indigofera parviflora

Heyne ex Hook. & Arn. 1834

##### Materials

- **Type status:** Other material. **Location:** continent: Asia; country: India; countryCode: IN; stateProvince: Maharashtra; municipality: Chakur; locality: Wagholi; verbatimLatitude: 18°34.266N; verbatimLongitude: 76°48.271E; verbatimCoordinateSystem: degrees minutes; geodeticDatum: WGS84; **Event:** month: August-January; fieldNumber: *RDG*- 1218; fieldNotes: Erect herbs; **Record Level:** institutionCode: Wachland College of Arts & Science, Solapur (WCAS).

#### Indigofera prostrata

Willd. 1802

##### Materials

- **Type status:** Other material. **Location:** continent: Asia; country: India; countryCode: IN; stateProvince: Maharashtra; municipality: Solapur; locality: Solapur district; **Event:** month: August-October; fieldNumber: *Acland*- 389; fieldNotes: Erect herbs; **Record Level:** institutionCode: Blatter Herbaium, St. Xavier’s College, Mumbai (BLAT).

#### Indigofera spicata

Forssk. 1775

##### Materials

- **Type status:** Other material. **Location:** continent: Asia; country: India; countryCode: IN; stateProvince: Maharashtra; municipality: Ausa; locality: Ashiv; verbatimLatitude: 18°06.077N; verbatimLongitude: 76°20.904E; verbatimCoordinateSystem: degrees minutes; geodeticDatum: WGS84; **Event:** month: August-December; fieldNumber: *RDG*- 1155; fieldNotes: Erect herbs; **Record Level:** institutionCode: Wachland College of Arts & Science, Solapur (WCAS).

#### Indigofera tinctoria

L. 1753

##### Materials

- **Type status:** Other material. **Location:** continent: Asia; country: India; countryCode: IN; stateProvince: Maharashtra; municipality: Patoda (Beed); locality: Sautada; verbatimLatitude: 18°48.564N; verbatimLongitude: 75°21.415E; verbatimCoordinateSystem: degrees minutes; geodeticDatum: WGS84; **Event:** month: August-January; fieldNumber: *RDG*- 226; fieldNotes: Shrubs; **Record Level:** institutionCode: Wachland College of Arts & Science, Solapur (WCAS).

#### Indigofera trifoliatavar.duthiei

(Drumm. ex Naik) Sanjappa, 1991

##### Materials

- **Type status:** Other material. **Location:** continent: Asia; country: India; countryCode: IN; stateProvince: Maharashtra; municipality: Latur; locality: Ausa; verbatimLatitude: 18°15.560N; verbatimLongitude: 76°30.998E; verbatimCoordinateSystem: degrees minutes; geodeticDatum: WGS84; **Event:** month: September-November; fieldNumber: *RDG*- 340; fieldNotes: Shrubs; **Record Level:** institutionCode: Wachland College of Arts & Science, Solapur (WCAS).

#### Indigofera trifoliatavar.trifoliata

L. 1756

##### Materials

- **Type status:** Other material. **Location:** continent: Asia; country: India; countryCode: IN; stateProvince: Maharashtra; municipality: Dharur; locality: Choramba; verbatimLatitude: 18°51.684N; verbatimLongitude: 76°05.462E; verbatimCoordinateSystem: degrees minutes; geodeticDatum: WGS84; **Event:** month: August-December; fieldNumber: *RDG-* 1226; fieldNotes: Erect herbs; **Record Level:** institutionCode: Wachland College of Arts & Science, Solapur (WCAS).

#### Indigofera trita

L. f. 1781

##### Materials

- **Type status:** Other material. **Location:** continent: Asia; country: India; countryCode: IN; stateProvince: Maharashtra; municipality: Ambajogai; locality: Murkatwadi; verbatimLatitude: 18°44.180N; verbatimLongitude: 76°35.262E; verbatimCoordinateSystem: degrees minutes; geodeticDatum: WGS84; **Event:** month: August-January; fieldNumber: *RDG*- 028; fieldNotes: Erect herbs; **Record Level:** institutionCode: Wachland College of Arts & Science, Solapur (WCAS).

#### Lablab purpureusvar.lignosus

(L.) King, 1898

##### Materials

- **Type status:** Other material. **Location:** continent: Asia; country: India; countryCode: IN; stateProvince: Maharashtra; municipality: Latur; locality: Nagzari; verbatimLatitude: 18°28.073N; verbatimLongitude: 76°30.744E; verbatimCoordinateSystem: degrees minutes; geodeticDatum: WGS84; **Event:** month: October-January; fieldNumber: *RDG*- 479; fieldNotes: Woody climbers; **Record Level:** institutionCode: Wachland College of Arts & Science, Solapur (WCAS).

#### Lablab purpureusvar.purpureus

(L.) Sweet, 1970

##### Materials

- **Type status:** Other material. **Location:** continent: Asia; country: India; countryCode: IN; stateProvince: Maharashtra; municipality: Ambajogai; locality: Talni; verbatimLatitude: 18°44.197N; verbatimLongitude: 76°30.808E; verbatimCoordinateSystem: degrees minutes; geodeticDatum: WGS84; **Event:** month: September-February; fieldNumber: *RDG*- 023; fieldNotes: Woody climbers; **Record Level:** institutionCode: Wachland College of Arts & Science, Solapur (WCAS).

#### Lathyrus sativus

L. 1753

##### Materials

- **Type status:** Other material. **Location:** continent: Asia; country: India; countryCode: IN; stateProvince: Maharashtra; municipality: Akkalkot (Solapur); locality: Hunoor; **Event:** month: December-February; fieldNumber: *RDG*- 1120; fieldNotes: Erect herbs; **Record Level:** institutionCode: Wachland College of Arts & Science, Solapur (WCAS).

#### Lens culinaris

Medik. 1788

##### Materials

- **Type status:** Other material. **Location:** continent: Asia; country: India; countryCode: IN; stateProvince: Maharashtra; municipality: Osmanabad; locality: Panchmahal (near Bembli); verbatimLatitude: 18°09.192N; verbatimLongitude: 76°11.897E; verbatimCoordinateSystem: degrees minutes; geodeticDatum: WGS84; **Event:** month: December-February; fieldNumber: *RDG*- 461; fieldNotes: Erect herbs; **Record Level:** institutionCode: Wachland College of Arts & Science, Solapur (WCAS).
- **Type status:** Other material. **Location:** continent: Asia; country: India; countryCode: IN; stateProvince: Maharashtra; municipality: Osmanabad; locality: Bembli; **Event:** month: December-February; fieldNumber: *R.D. Gore-* 13184; fieldNotes: Erect herbs; **Record Level:** institutionCode: Wachland College of Arts & Science, Solapur (WCAS).

#### Macroptilium lathyroidesvar.semierectum

(L.) Urb. 1928

##### Materials

- **Type status:** Other material. **Location:** continent: Asia; country: India; countryCode: IN; stateProvince: Maharashtra; municipality: Solapur; locality: Barshi town; verbatimLatitude: 18°14.544N; verbatimLongitude: 75°41.718E; verbatimCoordinateSystem: degrees minutes; geodeticDatum: WGS84; **Event:** month: September-December; fieldNumber: *RDG*- 664; fieldNotes: Woody climbers; **Record Level:** institutionCode: Wachland College of Arts & Science, Solapur (WCAS).

#### Macrotyloma uniflorum

(Lam.) Verdc. 1970

##### Materials

- **Type status:** Other material. **Location:** continent: Asia; country: India; countryCode: IN; stateProvince: Maharashtra; municipality: Patoda; locality: Chincholi; verbatimLatitude: 18°55.864N; verbatimLongitude: 75°15.331E; verbatimCoordinateSystem: degrees minutes; geodeticDatum: WGS84; **Event:** month: September-November; fieldNumber: *RDG*- 282; fieldNotes: Woody climbers; **Record Level:** institutionCode: Wachland College of Arts & Science, Solapur (WCAS).
- **Type status:** Other material. **Location:** continent: Asia; country: India; countryCode: IN; stateProvince: Maharashtra; municipality: Lohara; locality: Near Wadgaon fata; verbatimLatitude: 17°55.876N; verbatimLongitude: 76°20.938E; verbatimCoordinateSystem: degrees minutes; geodeticDatum: WGS84; **Event:** month: September-November; fieldNumber: *R.D. Gore-* 13151; fieldNotes: Woody climbers; **Record Level:** institutionCode: Wachland College of Arts & Science, Solapur (WCAS).

#### Medicago sativa

L. 1753

##### Materials

- **Type status:** Other material. **Location:** continent: Asia; country: India; countryCode: IN; stateProvince: Maharashtra; municipality: Ausa; locality: Kharosa; verbatimLatitude: 18°09.199N; verbatimLongitude: 76°40.085E; verbatimCoordinateSystem: degrees minutes; geodeticDatum: WGS84; **Event:** month: December-January; fieldNumber: *RDG*- 606; fieldNotes: Erect herbs; **Record Level:** institutionCode: Wachland College of Arts & Science, Solapur (WCAS).

#### Melilotus albus

Medik. 1787

##### Materials

- **Type status:** Other material. **Location:** continent: Asia; country: India; countryCode: IN; stateProvince: Maharashtra; municipality: Paranda; locality: Domgaon; verbatimLatitude: 18°18.939N; verbatimLongitude: 75°24.401E; verbatimCoordinateSystem: degrees minutes; geodeticDatum: WGS84; **Event:** month: January-August; fieldNumber: *RDG*- 636; fieldNotes: Erect herbs; **Record Level:** institutionCode: Wachland College of Arts & Science, Solapur (WCAS).

#### Melilotus indicus

(L.) All. 1785

##### Materials

- **Type status:** Other material. **Location:** continent: Asia; country: India; countryCode: IN; stateProvince: Maharashtra; municipality: Tuljapur; locality: Wanegaon; verbatimLatitude: 17°57.009N; verbatimLongitude: 76°12.329E; verbatimCoordinateSystem: degrees minutes; geodeticDatum: WGS84; **Event:** month: January-March; fieldNumber: *RDG*- 559; fieldNotes: Erect herbs; **Record Level:** institutionCode: Wachland College of Arts & Science, Solapur (WCAS).
- **Type status:** Other material. **Location:** continent: Asia; country: India; countryCode: IN; stateProvince: Maharashtra; municipality: Tuljapur; locality: Naldurg; verbatimLatitude: 17°49.405N; verbatimLongitude: 76°17.531E; verbatimCoordinateSystem: degrees minutes; geodeticDatum: WGS84; **Event:** month: January-March; fieldNumber: *R.D. Gore-* 13186; fieldNotes: Erect herbs; **Record Level:** institutionCode: Wachland College of Arts & Science, Solapur (WCAS).

#### Mucuna minima

Haines, 1919

##### Materials

- **Type status:** Other material. **Location:** continent: Asia; country: India; countryCode: IN; stateProvince: Maharashtra; municipality: Osmanabad; locality: Gad-devdari; verbatimLatitude: 18°15.828N; verbatimLongitude: 75°59.417E; verbatimCoordinateSystem: degrees minutes; geodeticDatum: WGS84; **Event:** month: October-January; fieldNumber: *RDG*- 1013; fieldNotes: Trailing/twining herbs; **Record Level:** institutionCode: Wachland College of Arts & Science, Solapur (WCAS).

#### Mucuna pruriens

(L.) DC. 1825

##### Materials

- **Type status:** Other material. **Location:** continent: Asia; country: India; countryCode: IN; stateProvince: Maharashtra; municipality: Osmanabad; locality: Wadgaon-Siddheshwar; verbatimLatitude: 18°07.566N; verbatimLongitude: 76°04.029E; verbatimCoordinateSystem: degrees minutes; geodeticDatum: WGS84; **Event:** month: October-December; fieldNumber: *RDG*- 872; fieldNotes: Woody climbers; **Record Level:** institutionCode: Wachland College of Arts & Science, Solapur (WCAS).

#### Neonotonia wightii

(Grah. ex Wight & Arn.) Lackey, 1977

##### Materials

- **Type status:** Other material. **Location:** continent: Asia; country: India; countryCode: IN; stateProvince: Maharashtra; county: Marathwada; locality: Beed district; **Event:** month: September-December; fieldNumber: *Naik*- 397; fieldNotes: Woody climbers; **Record Level:** institutionCode: Herbarium of Dr. Babasaheb Ambedkar Marathwada University, Aurangabad (BAMU).
- **Type status:** Other material. **Location:** continent: Asia; country: India; countryCode: IN; stateProvince: Maharashtra; county: Marathwada; locality: Osmanabad; **Event:** month: November; fieldNumber: *Almeida*- s.n.; fieldNotes: Woody climbers; **Record Level:** institutionCode: Blatter Herbarium, St. Xeviers College Mumbai (BLAT).

#### Paracalyx scariosus

(Roxb.) Ali, 1968

##### Materials

- **Type status:** Other material. **Location:** continent: Asia; country: India; countryCode: IN; stateProvince: Maharashtra; municipality: Osmanabad; locality: Ramling sanctuary; verbatimLatitude: 18°17.946N; verbatimLongitude: 75°56.773E; verbatimCoordinateSystem: degrees minutes; geodeticDatum: WGS84; **Event:** month: December-March; fieldNumber: *RDG*- 542; fieldNotes: Woody climbers; **Record Level:** institutionCode: Wachland College of Arts & Science, Solapur (WCAS).

#### Phaseolus lunatus

L. 1753

##### Materials

- **Type status:** Other material. **Location:** continent: Asia; country: India; countryCode: IN; stateProvince: Maharashtra; municipality: Lohara; locality: Lohara town; **Event:** month: November-February; fieldNumber: *RDG*- 1105; fieldNotes: Trailing/twining herb; **Record Level:** institutionCode: Wachland College of Arts & Science, Solapur (WCAS).

#### Phaseolus vulgaris

L. 1753

##### Materials

- **Type status:** Other material. **Location:** continent: Asia; country: India; countryCode: IN; stateProvince: Maharashtra; municipality: Tuljapur; locality: Apsinga; verbatimLatitude: 18°04.951N; verbatimLongitude: 76°01.676E; verbatimCoordinateSystem: degrees minutes; geodeticDatum: WGS84; **Event:** month: September-February; fieldNumber: *RSD*- 1210; fieldNotes: Trailing/twining herbs; **Record Level:** institutionCode: Wachland College of Arts & Science, Solapur (WCAS).

#### Pisum arvense

L. 1753

##### Materials

- **Type status:** Other material. **Location:** continent: Asia; country: India; countryCode: IN; stateProvince: Maharashtra; municipality: Omerga; locality: Madaj; verbatimLatitude: 17°55.902N; verbatimLongitude: 76°37.176E; verbatimCoordinateSystem: degrees minutes; geodeticDatum: WGS84; **Event:** month: September-February; fieldNumber: *RSD*- 019; fieldNotes: Trailing/twining herbs; **Record Level:** institutionCode: Wachland College of Arts & Science, Solapur (WCAS).

#### Pisum sativum

L. 1753

##### Materials

- **Type status:** Other material. **Location:** continent: Asia; country: India; countryCode: IN; stateProvince: Maharashtra; municipality: Tuljapur; locality: Sindphal; **Event:** month: August-February; fieldNumber: *RSD*- 120; fieldNotes: Trailing/twining herbs; **Record Level:** institutionCode: Wachland College of Arts & Science, Solapur (WCAS).

#### Pongamia pinnata

(L.) Pierre, 1899

##### Materials

- **Type status:** Other material. **Location:** continent: Asia; country: India; countryCode: IN; stateProvince: Maharashtra; municipality: Omerga; locality: Wagdari; verbatimLatitude: 17°55.167N; verbatimLongitude: 76°39.447E; verbatimCoordinateSystem: degrees minutes; geodeticDatum: WGS84; **Event:** month: March-August; fieldNumber: *RDG*- 576; fieldNotes: Trees; **Record Level:** institutionCode: Wachland College of Arts & Science, Solapur (WCAS).
- **Type status:** Other material. **Location:** continent: Asia; country: India; countryCode: IN; stateProvince: Maharashtra; municipality: Tuljapur; locality: Naldurg; verbatimLatitude: 17°49.366N; verbatimLongitude: 76°17.645E; verbatimCoordinateSystem: degrees minutes; geodeticDatum: WGS84; **Event:** month: March-August; fieldNumber: *R.D. Gore-* 13185; fieldNotes: Trees; **Record Level:** institutionCode: Wachland College of Arts & Science, Solapur (WCAS).

#### Pseudarthria viscida

(L.) Wight & Arn. 1834

##### Materials

- **Type status:** Other material. **Location:** continent: Asia; country: India; countryCode: IN; stateProvince: Maharashtra; municipality: Osmanabad; locality: Papnas; verbatimLatitude: 18°09.370N; verbatimLongitude: 76°03.071E; verbatimCoordinateSystem: degrees minutes; geodeticDatum: WGS84; **Event:** month: August-December; fieldNumber: *RDG*- 186; fieldNotes: Shrubs; **Record Level:** institutionCode: Wachland College of Arts & Science, Solapur (WCAS).

#### Psophocarpus tetragonolobus

(L.) DC. 1825

##### Materials

- **Type status:** Other material. **Location:** continent: Asia; country: India; countryCode: IN; stateProvince: Maharashtra; municipality: Pathardi (Ahmednagar); locality: Dhakanwadi; verbatimLatitude: 18°59.782N; verbatimLongitude: 75°18.645E; verbatimCoordinateSystem: degrees minutes; geodeticDatum: WGS84; **Event:** month: October-March; fieldNumber: *RDG*- s.n.; fieldNotes: Trailing/twining herbs; **Record Level:** institutionCode: Wachland College of Arts & Science, Solapur (WCAS).

#### Pterocarpus marsupium

Roxb. 1799

##### Materials

- **Type status:** Other material. **Location:** continent: Asia; country: India; countryCode: IN; stateProvince: Maharashtra; municipality: Ambajogai; locality: Talni; verbatimLatitude: 18°45.211N; verbatimLongitude: 76°30.800E; verbatimCoordinateSystem: degrees minutes; geodeticDatum: WGS84; **Event:** month: December-March; fieldNumber: *RDG*- 016; fieldNotes: Trees; **Record Level:** institutionCode: Wachland College of Arts & Science, Solapur (WCAS).
- **Type status:** Other material. **Location:** continent: Asia; country: India; countryCode: IN; stateProvince: Maharashtra; municipality: Tuljapur; locality: Apsinga; verbatimLatitude: 18°04.047N; verbatimLongitude: 76°04.035E; verbatimCoordinateSystem: degrees minutes; geodeticDatum: WGS84; **Event:** month: December-March; fieldNumber: *R.D. Gore-* 13102; fieldNotes: Trees; **Record Level:** institutionCode: Wachland College of Arts & Science, Solapur (WCAS).

#### Pueraria montanavar.lobata

(Willd.) Sanjappa, 1991

##### Materials

- **Type status:** Other material. **Location:** continent: Asia; country: India; countryCode: IN; stateProvince: Maharashtra; municipality: Tuljapur; locality: Pandhar (Apsinga); verbatimLatitude: 18°04.140N; verbatimLongitude: 76°02.000E; verbatimCoordinateSystem: degrees minutes; geodeticDatum: WGS84; **Event:** month: January-June; fieldNumber: *RDG*- 725; fieldNotes: Woody climbers; **Record Level:** institutionCode: Wachland College of Arts & Science, Solapur (WCAS).

#### Rhynchosia capitata

(Heyne ex Roth) DC. 1825

##### Materials

- **Type status:** Other material. **Location:** continent: Asia; country: India; countryCode: IN; stateProvince: Maharashtra; municipality: Tuljapur; locality: Jalkot (Naldurg); verbatimLatitude: 17°49.654N; verbatimLongitude: 76°19.680E; verbatimCoordinateSystem: degrees minutes; geodeticDatum: WGS84; **Event:** month: August-October; fieldNumber: *RDG*- 364; fieldNotes: Trailing/twining herbs; **Record Level:** institutionCode: Wachland College of Arts & Science, Solapur (WCAS).

#### Rhynchosia minimavar.laxiflora

(Camb.) Baker, 1876

##### Materials

- **Type status:** Other material. **Location:** continent: Asia; country: India; countryCode: IN; stateProvince: Maharashtra; municipality: Dharur (Beed); locality: Dharur fort; verbatimLatitude: 18°49.142N; verbatimLongitude: 76°06.278E; verbatimCoordinateSystem: degrees minutes; geodeticDatum: WGS84; **Event:** month: June-November; fieldNumber: *RDG*- 177; fieldNotes: Woody climbers; **Record Level:** institutionCode: Wachland College of Arts & Science, Solapur (WCAS).

#### Rhynchosia minimavar.minima

(L.) DC. 1825

##### Materials

- **Type status:** Other material. **Location:** continent: Asia; country: India; countryCode: IN; stateProvince: Maharashtra; municipality: Kalamb; locality: Awad-Shirpura; verbatimLatitude: 18°34.159N; verbatimLongitude: 76°11.167E; verbatimCoordinateSystem: degrees minutes; geodeticDatum: WGS84; **Event:** month: July-December; fieldNumber: *RDG*- 124; fieldNotes: Woody climbers; **Record Level:** institutionCode: Wachland College of Arts & Science, Solapur (WCAS).

#### Rhynchosia rothii

Benth. ex Ait. 1869

##### Materials

- **Type status:** Other material. **Location:** continent: Asia; country: India; countryCode: IND; stateProvince: Maharashtra; municipality: Patoda; locality: Sautada; verbatimLatitude: 18°47.804N; verbatimLongitude: 75°21.043E; verbatimCoordinateSystem: degrees minutes; geodeticDatum: WGS84; **Event:** month: September-November; fieldNumber: *RDG*- 1000; fieldNotes: Woody climbers; **Record Level:** institutionCode: Wachland College of Arts & Science, Solapur (WCAS).

#### Sesbania bispinosa

(Jacq.) Steud. ex Wight, 1909

##### Materials

- **Type status:** Other material. **Location:** continent: Asia; country: India; countryCode: IN; stateProvince: Maharashtra; municipality: Beed; locality: Dharur; verbatimLatitude: 18°48.359N; verbatimLongitude: 76°06.298E; verbatimCoordinateSystem: degrees minutes; geodeticDatum: WGS84; **Event:** month: September-November; fieldNumber: *RDG*- 400; fieldNotes: Erect herbs; **Record Level:** institutionCode: Wachland College of Arts & Science, Solapur (WCAS).

#### Sesbania cannabina

(Retz.) Poir. 1806

##### Materials

- **Type status:** Other material. **Location:** continent: Asia; country: India; countryCode: IN; stateProvince: Maharashtra; municipality: Ashti (Beed); locality: Karanji; **Event:** month: August-January; fieldNumber: *RDG*- 1191; fieldNotes: Erect herbs; **Record Level:** institutionCode: Wachland College of Arts & Science, Solapur (WCAS).

#### Sesbania grandiflora

(L.) Poir. 1806

##### Materials

- **Type status:** Other material. **Location:** continent: Asia; country: India; countryCode: IN; stateProvince: Maharashtra; municipality: Osmanabad; locality: Bembli; **Event:** month: September-April; fieldNumber: *RDG*- 805; fieldNotes: Trees; **Record Level:** institutionCode: Wachland College of Arts & Science, Solapur (WCAS).
- **Type status:** Other material. **Location:** continent: Asia; country: India; countryCode: IN; stateProvince: Maharashtra; municipality: Tuljapur; locality: Near Tamalwadi; verbatimLatitude: 17°50.842N; verbatimLongitude: 75°57.829E; verbatimCoordinateSystem: degrees minutes; geodeticDatum: WGS84; **Event:** month: September-April; fieldNumber: *R.D. Gore-* 13173; fieldNotes: Trees; **Record Level:** institutionCode: Wachland College of Arts & Science, Solapur (WCAS).

#### Sesbania sesban

(L.) Merr. 1912

##### Materials

- **Type status:** Other material. **Location:** continent: Asia; country: India; countryCode: IN; stateProvince: Maharashtra; municipality: Patoda; locality: Matwali; verbatimLatitude: 18°48.496N; verbatimLongitude: 75°18.005E; verbatimCoordinateSystem: degrees minutes; geodeticDatum: WGS84; **Event:** month: December-March; fieldNumber: *RDG*- 911; fieldNotes: Shrubs; **Record Level:** institutionCode: Wachland College of Arts & Science, Solapur (WCAS).

#### Stylosanthes fruticosa

(Retz.) Alst. 1931

##### Materials

- **Type status:** Other material. **Location:** continent: Asia; country: India; countryCode: IN; stateProvince: Maharashtra; municipality: Chakur; locality: Mandurki; verbatimLatitude: 18°28.950N; verbatimLongitude: 76°54.276E; verbatimCoordinateSystem: degrees minutes; geodeticDatum: WGS84; **Event:** month: September-March; fieldNumber: *RDG*- 039; fieldNotes: Shrubs; **Record Level:** institutionCode: Wachland College of Arts & Science, Solapur (WCAS).

#### Stylosanthes hamata

(L.) Taub. 1890

##### Materials

- **Type status:** Other material. **Location:** continent: Asia; country: India; countryCode: IN; stateProvince: Maharashtra; municipality: Patoda; locality: Matwali; verbatimLatitude: 18°48.729N; verbatimLongitude: 75°17.970E; verbatimCoordinateSystem: degrees minutes; geodeticDatum: WGS84; **Event:** month: August-February; fieldNumber: *RDG*- 919; fieldNotes: Erect herbs; **Record Level:** institutionCode: Wachland College of Arts & Science, Solapur (WCAS).

#### Taverniera cuneifolia

(Roth.) Arn. 1836

##### Materials

- **Type status:** Other material. **Location:** continent: Asia; country: India; countryCode: IN; stateProvince: Maharashtra; municipality: Tuljapur; locality: Apsinga; verbatimLatitude: 18°04.849N; verbatimLongitude: 76°01.712E; verbatimCoordinateSystem: degrees minutes; geodeticDatum: WGS84; **Event:** month: December-February; fieldNumber: *RDG*- 042; fieldNotes: Shrubs; **Record Level:** institutionCode: Wachland College of Arts & Science, Solapur (WCAS).

#### Tephrosia hamiltonii

Drum. 1918

##### Materials

- **Type status:** Other material. **Location:** continent: Asia; country: India; countryCode: IN; stateProvince: Maharashtra; municipality: Jamkhed; locality: Shirur; verbatimLatitude: 18°43.591N; verbatimLongitude: 75°22.370E; verbatimCoordinateSystem: degrees minutes; geodeticDatum: WGS84; **Event:** month: July-January; fieldNumber: *RDG*- 1099; fieldNotes: Shrubs; **Record Level:** institutionCode: Wachland College of Arts & Science, Solapur (WCAS).

#### Tephrosia leptostachya

DC. 1825

##### Materials

- **Type status:** Other material. **Location:** continent: Asia; country: India; countryCode: IN; stateProvince: Maharashtra; municipality: Tuljapur; locality: Kawaldara; verbatimLatitude: 18°03.865N; verbatimLongitude: 76°05.590E; verbatimCoordinateSystem: degrees minutes; geodeticDatum: WGS84; **Event:** month: July-January; fieldNumber: *RDG*- 663; fieldNotes: Erect herbs; **Record Level:** institutionCode: Wachland College of Arts & Science, Solapur (WCAS).

#### Tephrosia pumila

(Lam.) Pers. 1807

##### Materials

- **Type status:** Other material. **Location:** continent: Asia; country: India; countryCode: IN; stateProvince: Maharashtra; municipality: Ambajogai; locality: Talni; verbatimLatitude: 18°45.287N; verbatimLongitude: 76°30.729E; verbatimCoordinateSystem: degrees minutes; geodeticDatum: WGS84; **Event:** month: September-February; fieldNumber: *RDG*- 017; fieldNotes: Erect herbs; **Record Level:** institutionCode: Wachland College of Arts & Science, Solapur (WCAS).

#### Tephrosia purpurea

(L.) Pers. 1807

##### Materials

- **Type status:** Other material. **Location:** continent: Asia; country: India; countryCode: IN; stateProvince: Maharashtra; municipality: Tuljapur; locality: Apsinga; verbatimLatitude: 18°035.17N; verbatimLongitude: 76°30.14E; verbatimCoordinateSystem: degrees minutes; geodeticDatum: WGS84; **Event:** month: August-December; fieldNumber: **​R.D.* Gore*- 13074; fieldNotes: Erect herbs; **Record Level:** institutionCode: Wachland College of Arts & Science, Solapur (WCAS).

#### Tephrosia senticosa

(L.) Pers. 1807

##### Materials

- **Type status:** Other material. **Location:** continent: Asia; country: India; countryCode: IN; stateProvince: Maharashtra; municipality: Pathardi; locality: Chinchpur; verbatimLatitude: 19°00.506N; verbatimLongitude: 75°18.188E; verbatimCoordinateSystem: degrees minutes; geodeticDatum: WGS84; **Event:** month: September-November; fieldNumber: *RDG*- 367; fieldNotes: Shrubs; **Record Level:** institutionCode: Wachland College of Arts & Science, Solapur (WCAS).

#### Tephrosia strigosa

(Dalz.) Santapau & Mahesh, 1957

##### Materials

- **Type status:** Other material. **Location:** continent: Asia; country: India; countryCode: IN; stateProvince: Maharashtra; municipality: Ambajogai; locality: Ghatnandur; verbatimLatitude: 18°44.095N; verbatimLongitude: 76°33.291E; verbatimCoordinateSystem: degrees minutes; geodeticDatum: WGS84; **Event:** month: August-September; fieldNumber: *RDG*- 012; fieldNotes: Erect herbs; **Record Level:** institutionCode: Wachland College of Arts & Science, Solapur (WCAS).

#### Tephrosia villosa

(L.) Pers. 1807

##### Materials

- **Type status:** Other material. **Location:** continent: Asia; country: India; countryCode: IN; stateProvince: Maharashtra; municipality: Kalamb; locality: Yermala; verbatimLatitude: 18°22.308N; verbatimLongitude: 75°51.904E; verbatimCoordinateSystem: degrees minutes; geodeticDatum: WGS84; **Event:** month: August-December; fieldNumber: *RDG*- 502; fieldNotes: Shrubs; **Record Level:** institutionCode: Wachland College of Arts & Science, Solapur (WCAS).

#### Teramnus labialis

(L. f.) Spreng. 1826

##### Materials

- **Type status:** Other material. **Location:** continent: Asia; country: India; countryCode: IN; stateProvince: Maharashtra; municipality: Tuljapur; locality: Apsinga; verbatimLatitude: 18°03.719N; verbatimLongitude: 76°02.903E; verbatimCoordinateSystem: degrees minutes; geodeticDatum: WGS84; **Event:** month: October-February; fieldNumber: *RDG*- 040; fieldNotes: Woody climbers; **Record Level:** institutionCode: Wachland College of Arts & Science, Solapur (WCAS).

#### Trigonella foenum-graecum

L. 1753

##### Materials

- **Type status:** Other material. **Location:** continent: Asia; country: India; countryCode: IN; stateProvince: Maharashtra; municipality: Ambajogai; locality: Talni; verbatimLatitude: 18°44.120N; verbatimLongitude: 76°30.854E; verbatimCoordinateSystem: degrees minutes; geodeticDatum: WGS84; **Event:** month: January-December; fieldNumber: *RDG*- 024; fieldNotes: Erect herbs; **Record Level:** institutionCode: Wachland College of Arts & Science, Solapur (WCAS).
- **Type status:** Other material. **Location:** continent: Asia; country: India; countryCode: IN; stateProvince: Maharashtra; municipality: Tuljapur; locality: Apsinga; verbatimLatitude: 18°04.951N; verbatimLongitude: 76°01.676E; verbatimCoordinateSystem: degrees minutes; geodeticDatum: WGS84; **Event:** month: January-December; fieldNumber: *R.D. Gore-* 13027; fieldNotes: Erect herbs; **Record Level:** institutionCode: Wachland College of Arts & Science, Solapur (WCAS).

#### Trigonella occulta

Del. 1812

##### Materials

- **Type status:** Other material. **Location:** continent: Asia; country: India; countryCode: IN; stateProvince: Maharashtra; municipality: Tuljapur; locality: Near Sindphal (Ghatshil); verbatimLatitude: 18°00.332N; verbatimLongitude: 76°03.837E; verbatimCoordinateSystem: degrees minutes; geodeticDatum: WGS84; **Event:** month: October-March; fieldNumber: *RDG*- 529; fieldNotes: Erect herbs; **Record Level:** institutionCode: Wachland College of Arts & Science, Solapur (WCAS).

#### Vigna aconitifolia

(Jacq.) Marechal, 1969

##### Materials

- **Type status:** Other material. **Location:** continent: Asia; country: India; countryCode: IN; stateProvince: Maharashtra; municipality: Nilanga; locality: Madansuri; verbatimLatitude: 18°01.649N; verbatimLongitude: 76°42.618E; verbatimCoordinateSystem: degrees minutes; geodeticDatum: WGS84; **Event:** month: August-November; fieldNumber: *RDG*- 232; fieldNotes: Trailing/twining herbs; **Record Level:** institutionCode: Wachland College of Arts & Science, Solapur (WCAS).

#### Vigna angularis

(Willd.) Ohwi. & Oha Shrubi, 1969

##### Materials

- **Type status:** Other material. **Location:** continent: Asia; country: India; countryCode: IN; stateProvince: Maharashtra; municipality: Nilanga; locality: Kasar-Sirshi; **Event:** month: August-November; fieldNumber: *RDG*- 1081; fieldNotes: Trailing/twining herbs; **Record Level:** institutionCode: Wachland College of Arts & Science, Solapur (WCAS).

#### Vigna indica

Dixit, Bhat & Yadav, 2011

##### Materials

- **Type status:** Other material. **Location:** continent: Asia; country: India; countryCode: IN; stateProvince: Maharashtra; municipality: Washi; locality: Kunthalgiri; verbatimLatitude: 18°32.666N; verbatimLongitude: 75°42.109E; verbatimCoordinateSystem: degrees minutes; geodeticDatum: WGS84; **Event:** month: August-January; fieldNumber: *RDG*- 233; fieldNotes: Erect herbs; **Record Level:** institutionCode: Wachland College of Arts & Science, Solapur (WCAS).

#### Vigna mungo

(L.) Hepper, 1956

##### Materials

- **Type status:** Other material. **Location:** continent: Asia; country: India; countryCode: IN; stateProvince: Maharashtra; municipality: Akkalkot; locality: Kajikanbas; **Event:** month: August-October; fieldNumber: *RDG*- 1077; fieldNotes: Erect herbs; **Record Level:** institutionCode: Wachland College of Arts & Science, Solapur (WCAS).

#### Vigna radiata

(L.) R. Wilczek, 1954

##### Materials

- **Type status:** Other material. **Location:** continent: Asia; country: India; countryCode: IN; stateProvince: Maharashtra; municipality: Nilanga; locality: Tambala; verbatimLatitude: 17°55.829N; verbatimLongitude: 76°54.044E; verbatimCoordinateSystem: degrees minutes; geodeticDatum: WGS84; **Event:** month: August-October; fieldNumber: *RSD*- 180; fieldNotes: Erect herbs; **Record Level:** institutionCode: Wachland College of Arts & Science, Solapur (WCAS).

#### Vigna stipulacea

Kuntze, 1891

##### Materials

- **Type status:** Other material. **Location:** continent: Asia; country: India; countryCode: IN; stateProvince: Maharashtra; municipality: Osmanabad; locality: Papnas; verbatimLatitude: 18°09.611N; verbatimLongitude: 76°03.049E; verbatimCoordinateSystem: degrees minutes; geodeticDatum: WGS84; **Event:** month: August-December; fieldNumber: *​R.D. Gore*- 13161; fieldNotes: Erect herbs; **Record Level:** institutionCode: Wachland College of Arts & Science, Solapur (WCAS).

#### Vigna sublobata

(Roxb.) Babu & Shrubarma, 1987

##### Materials

- **Type status:** Other material. **Location:** continent: Asia; country: India; countryCode: IN; stateProvince: Maharashtra; municipality: Washi; locality: Kunthalgiri; verbatimLatitude: 18°32.097N; verbatimLongitude: 75°41.563E; verbatimCoordinateSystem: degrees minutes; geodeticDatum: WGS84; **Event:** month: August-November; fieldNumber: *RDG*- 700; fieldNotes: Trailing/twining herbs; **Record Level:** institutionCode: Wachland College of Arts & Science, Solapur (WCAS).

#### Vigna trilobata

(L.) Verdc. 1968

##### Materials

- **Type status:** Other material. **Location:** continent: Asia; country: India; countryCode: IN; stateProvince: Maharashtra; municipality: Patoda (Beed); locality: Chincholi; verbatimLatitude: 18°55.914N; verbatimLongitude: 75°14.981E; verbatimCoordinateSystem: degrees minutes; geodeticDatum: WGS84; **Event:** month: August-December; fieldNumber: *RDG*- 284; fieldNotes: Erect herbs; **Record Level:** institutionCode: Wachland College of Arts & Science, Solapur (WCAS).

#### Vigna umbellata

(Thunb.) Ohwi & OhaShrubi, 1969

##### Materials

- **Type status:** Other material. **Location:** continent: Asia; country: India; countryCode: IN; stateProvince: Maharashtra; municipality: Washi; locality: Dindori; verbatimLatitude: 18°27.077N; verbatimLongitude: 75°46.480E; verbatimCoordinateSystem: degrees minutes; geodeticDatum: WGS84; **Event:** month: September-October; fieldNumber: *RDG*- 731; fieldNotes: Trailing/twining herbs; **Record Level:** institutionCode: Wachland College of Arts & Science, Solapur (WCAS).

#### Vigna unguiculata cylindrica

(L.) Eseltine, 1931

##### Materials

- **Type status:** Other material. **Location:** continent: Asia; country: India; countryCode: IN; stateProvince: Maharashtra; municipality: Pathardi (Ahmednagar); locality: Dhakanwadi; verbatimLatitude: 18°59.782N; verbatimLongitude: 75°18.645E; verbatimCoordinateSystem: degrees minutes; geodeticDatum: WGS84; **Event:** month: July-October; fieldNumber: *RDG*- 380; fieldNotes: Trailing/twining herbs; **Record Level:** institutionCode: Wachland College of Arts & Science, Solapur (WCAS).

#### Vigna unguiculata unguiculata

(L.) Walp. 1842

##### Materials

- **Type status:** Other material. **Location:** continent: Asia; country: India; countryCode: IN; stateProvince: Maharashtra; municipality: Tuljapur; locality: Near Devsinga; verbatimLatitude: 17°56.436N; verbatimLongitude: 76°10.496E; verbatimCoordinateSystem: degrees minutes; geodeticDatum: WGS84; **Event:** month: June-October; fieldNumber: *RSD*- 012; fieldNotes: Trailing/twining herbs; **Record Level:** institutionCode: Botanical Survey of India, Pune (BSI).

#### Zornia diphylla

(L.) Pers. 1807

##### Materials

- **Type status:** Other material. **Location:** continent: Asia; country: India; countryCode: IN; stateProvince: Maharashtra; municipality: Bhoom; locality: Shekapur (Ashta); verbatimLatitude: 18°23.935N; verbatimLongitude: 75°37.858E; verbatimCoordinateSystem: degrees minutes; geodeticDatum: WGS84; **Event:** month: July-October; fieldNumber: *RDG*- 149; fieldNotes: Erect herbs; **Record Level:** institutionCode: Wachland College of Arts & Science, Solapur (WCAS).

## Analysis

In the present work, authors have provided the first comprehensive inventory of legumes of Balaghat Ranges of Maharashtra. About 123 species, four subspecies and 17 varieties belonging to 83 genera of Fabaceae have been recorded from Balaghat Ranges of Maharashtra. The members of Fabaceae are dominant in herbaceous vegetation of the region. They are more diverse in genera like *Crotalaria* (23 taxa), *Indigofera* (16 taxa), *Alysicarpus* (14 taxa), *Vigna* (11 taxa) and *Desmodium* (8 taxa). Out of 144 leguminous taxa, 91 are herbs, 16 woody climbers or lianas, 24 shrubs and 13 trees (Table 1). Twelve taxa are endemic to India of which *Indigofera deccanensis* falls into Critically Endangered IUCN Red list category (Mishra and Singh 2001​).

## Discussion

The present study is an outcome of intensive and extensive field explorations and herbarium studies carried out between the years 2009-2013. The legumes of Balaghat Ranges show wide range of species diversity and growth forms. The total number of leguminous taxa reported for the Maharashtra State as on today is 344 (Almeida 1998; Kothari 2000; Lakshminarasimhan 2001). For Balaghat Ranges, the number of leguminous taxa works out to be 144. Therefore, percentage of leguminous taxa of Balaghat Ranges out of whole legumes of Maharashtra will be 41.86%. The members of Fabaceae are dominant in herbaceous vegetation and more speciose in genera like *Crotalaria* (23 taxa), *Indigofera* (16 taxa), *Alysicarpus* (14 taxa), *Vigna* (11 taxa) and *Desmodium* (8 taxa). The area under investigation is relatively small for the assessment of endemism of leguminous elements. It is continuous with the surrounding areas on the Deccan plateau and there is no obvious natural boundary between this and neighboring region, which can hold any endemic taxa. On the other hand, comparative statistical analysis of the leguminous species recorded so far from Balaghat Ranges with those of the neighboring regions reveals highest proportion of similarities. However, *Alysicarpus luteo-vexillatus*, A. pubescens var. vasavadae, *Crotalaria decasperma*, *C. filipes*, *C. hirta*, *C. leptostachya*, *C. linifolia*, *C. notonii*, *Indigofera deccanensis*, I. glandulosa var. sykesii, I. trifoliata var. duthiei and *Vigna indica* occur in Balaghat Ranges that are endemic to India. Among 12 endemic species, *Indigofera deccanensis* is known only from two localities with 907 Km^2^ extent of occurrence, 10 Km^2^ area of occupancy and severely fragmented small subpopulations. Hence, it falls into Critically Endangered IUCN Red List category (Mishra and Singh 2001; Gaikwad et al. 2014b​).

The legumes of Balaghat Ranges have many actual and potential uses such as food, fodder, industrial lubricants, natural dyes, medicine and sources of edible oil and timber; and indirectly affect socio-economic development of the region. Some nine legumes are used as vegetables in Balaghat Ranges that include *Canavalia gladiata*, *Cyamopsis tetragonolobus*, *Lablab purpureus*, *Phaseolus vulgaris*, *Pisum sativum*, *Pisum arvense*, *Sesbania grandiflora*, *Trigonella foenum-graecum*, Vigna unguiculata var. cylindrica and eight legumes namely *Cajanus cajan*, *Cicer arietinum*, *Lathyrus sativus*, *Lens culinaris*, *Macrotyloma uniflorum*, *Vigna aconitifolia*, *V. mungo* and *Vigna radiata* are cultivated as pulses. *Arachis hypogeal* and *Glycine max* are popular sources of edible oil. *Medicago sativa* and *Stylosanthes hamata* are cultivated as forage crops. *Dalbergia sissoo*, *D. melanoxylon*, *D. latifolium* and *Pterocarpus marsupium* are known for their quality timber whereas *Butea monosperma*, *Gliricidia sepium* and *Erythrina suberosa* are used as fuel wood. Flowers of *Butea monosperma* and *Indigofera tinctoria* are famous sources of natural dyes; later species is cultivated on commercial scale for yielding Indigo-dye. *Crotalaria juncea*, *Melilotus albus* and *M. indicus* are best green manures used by farmers. Seeds of *Pongamia pinnata* are a challenging source of non-edible oil, which has commercial importance as industrial lubricant. A reputed biofertilizer is prepared from seed of *Pongamia pinnata*. About 14 legumes of Balaghat Ranges have medicinal potential that include *Abrus precatorius*, *Butea monosperma*, Clitoria ternatea var. pilosula, C. ternatea var. ternatea, *Crotalaria verrucosa*, *Cullen corylifolia*, *Desmodium oojeinense*, *Mucuna pruriens*, *Pongamia pinnata*, *Pterocarpus marsupium*, *Tephrosia purpurea*, *Taverniera cuneifolia* and *Trigonella foenum-graecum*. About 19 leguminous taxa are wild relatives of our food and fodder crops have resistance to pests and diseases, and abiotic stresses such as drought and salinity can be used in improvement of the quality and yield of crops (Table 1).

## Figures and Tables

**Figure 2a.**
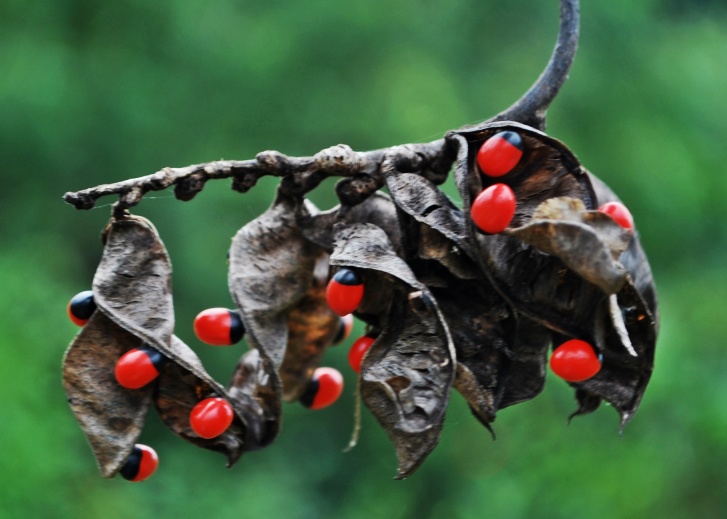
*Abrus precatorius* L.

**Figure 2b.**
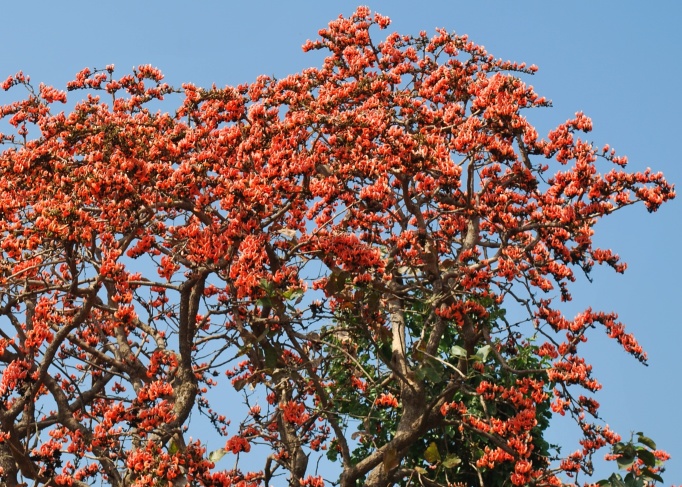
Butea monosperma (Lam.) Taub. var. monosperma

**Figure 2c.**
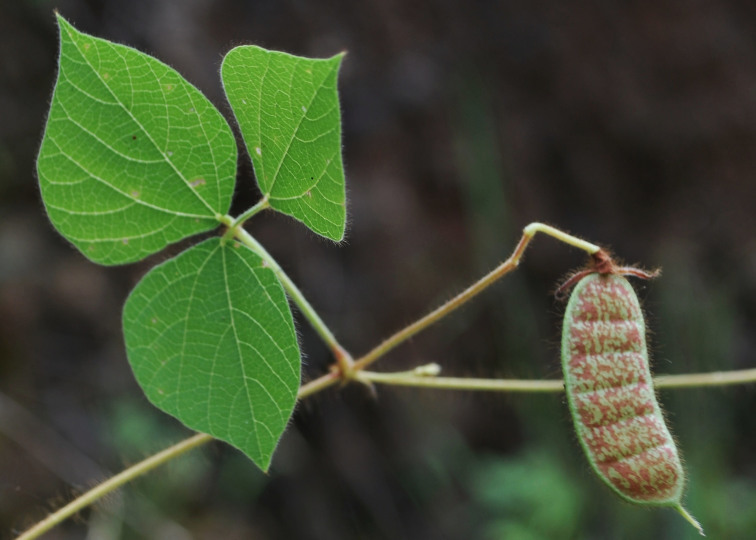
*Cajanus platycarpus* (Benth.) van der Maesen

**Figure 2d.**
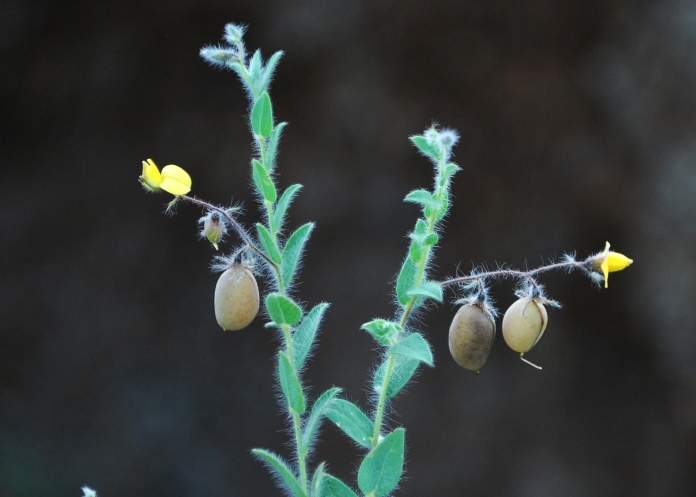
*Crotalaria filipes* Benth.

**Figure 2e.**
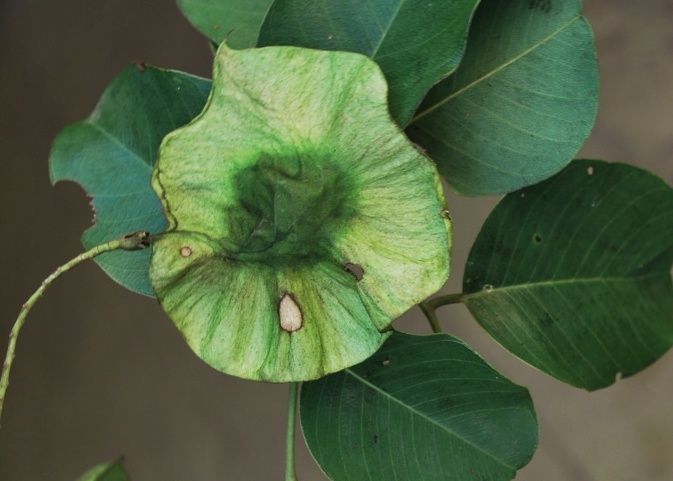
*Pterocarpus marsupium* Roxb.

**Figure 2f.**
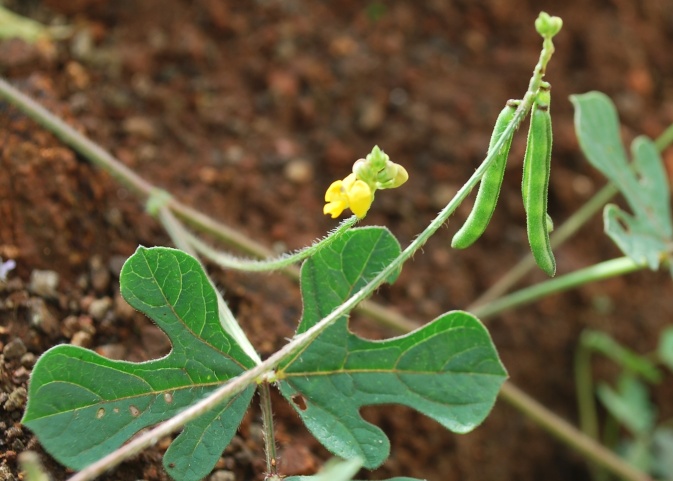
*Vigna indica* Dixit *et al.*

**Figure 3a.**
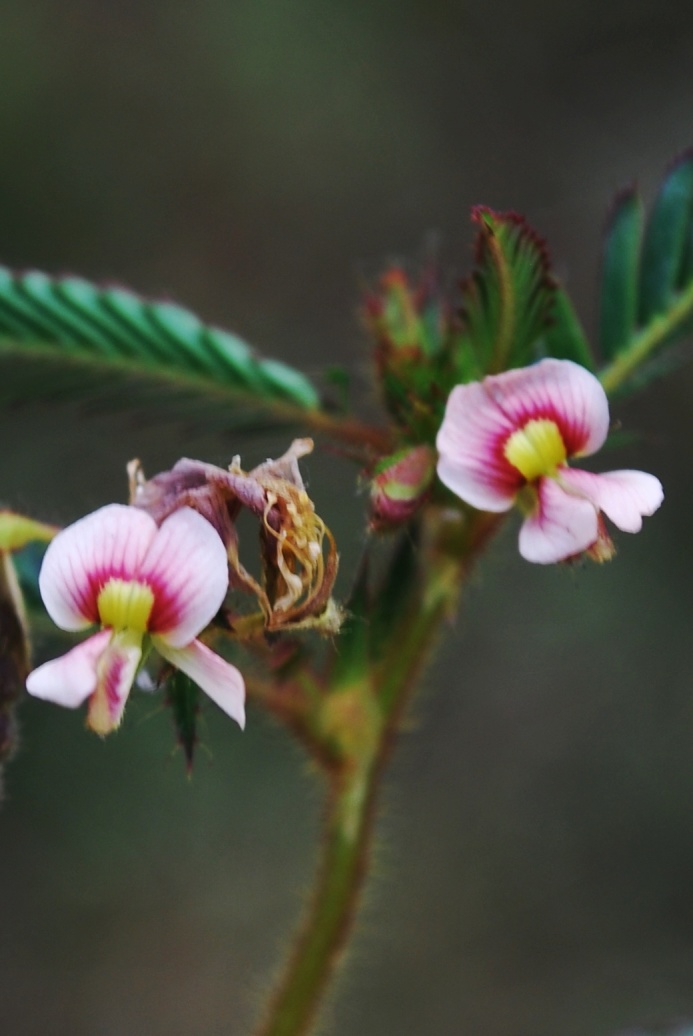
*Aeschynomene americana* L.

**Figure 3b.**
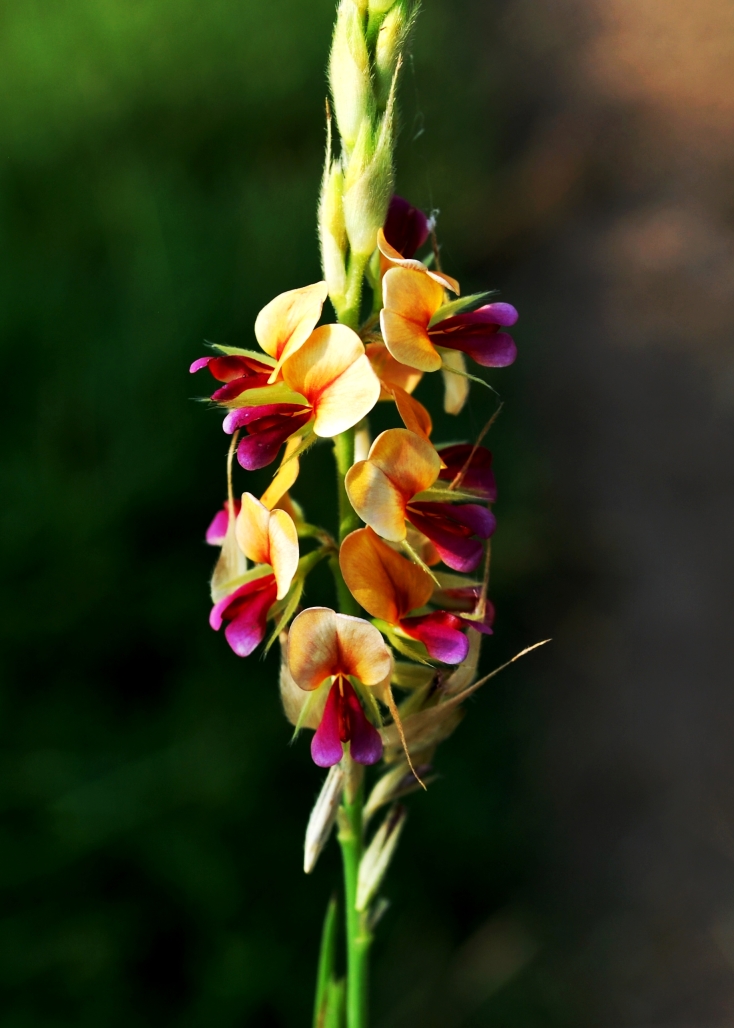
*Alysicarpus longifolius* Wight & Arn.

**Figure 3c.**
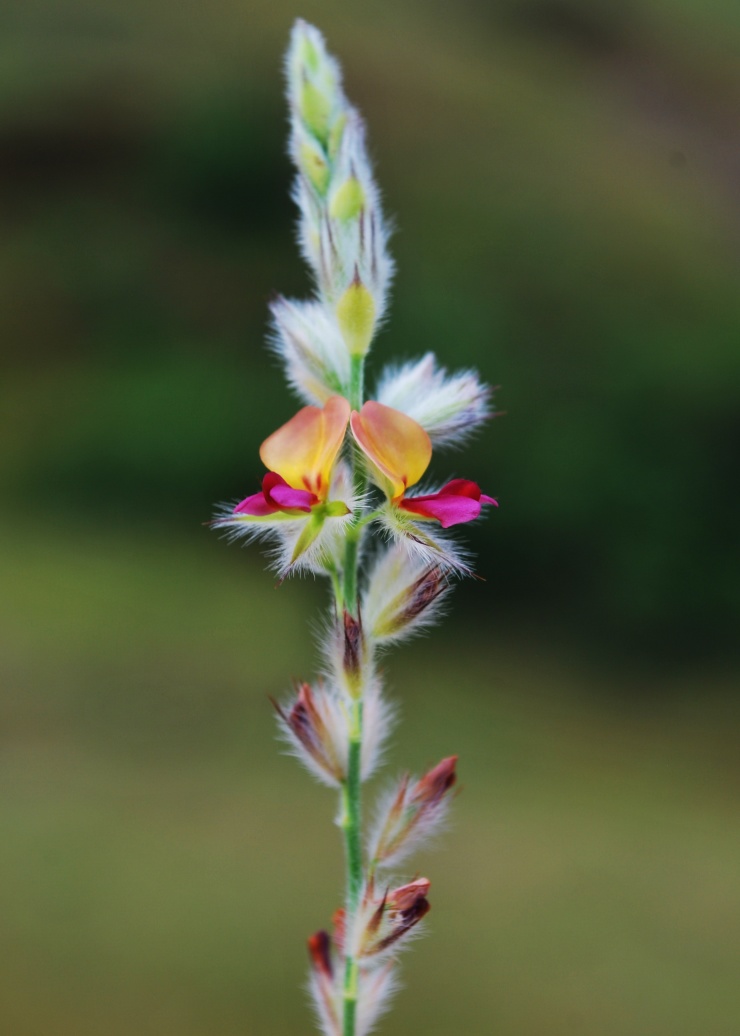
Alysicarpus pubescens Law var. vasavadae (Hemadri) Sanjappa

**Figure 3d.**
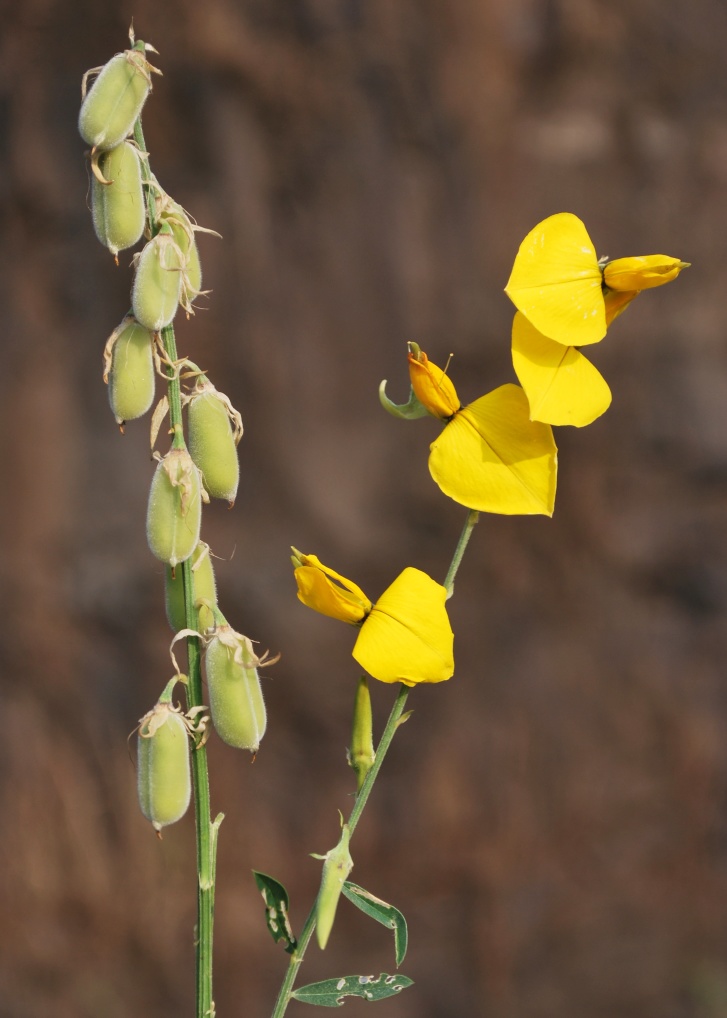
*Crotalaria leptostachya* Benth.

**Figure 3e.**
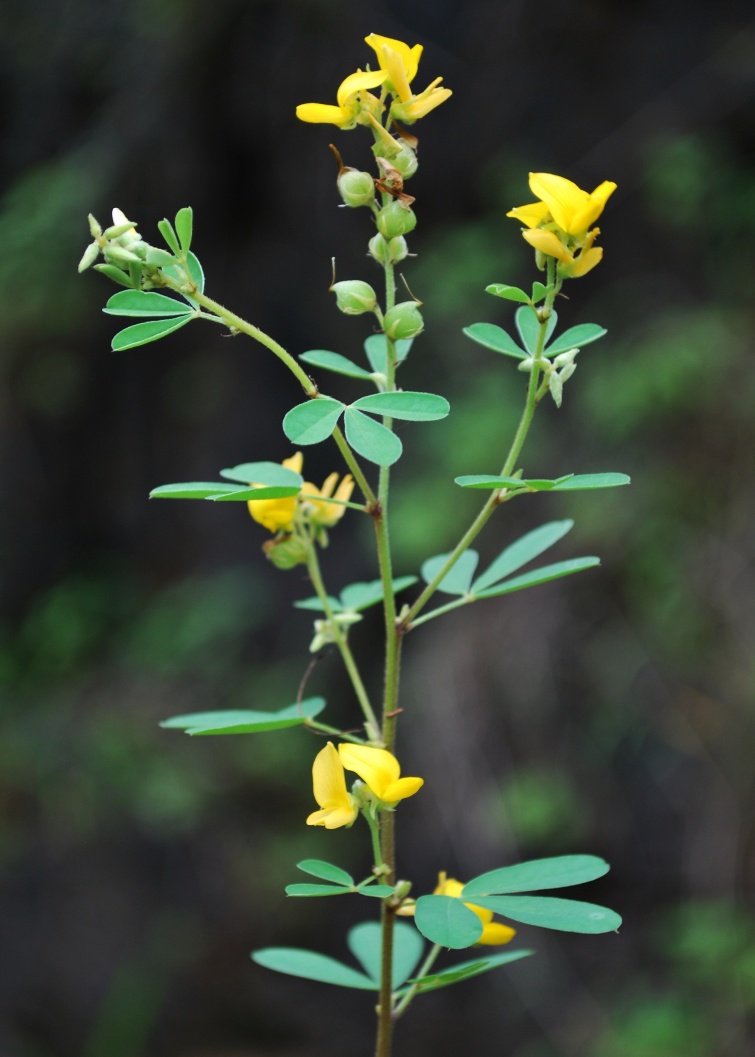
*Crotalaria notonii* Wight & Arn.

**Figure 3f.**
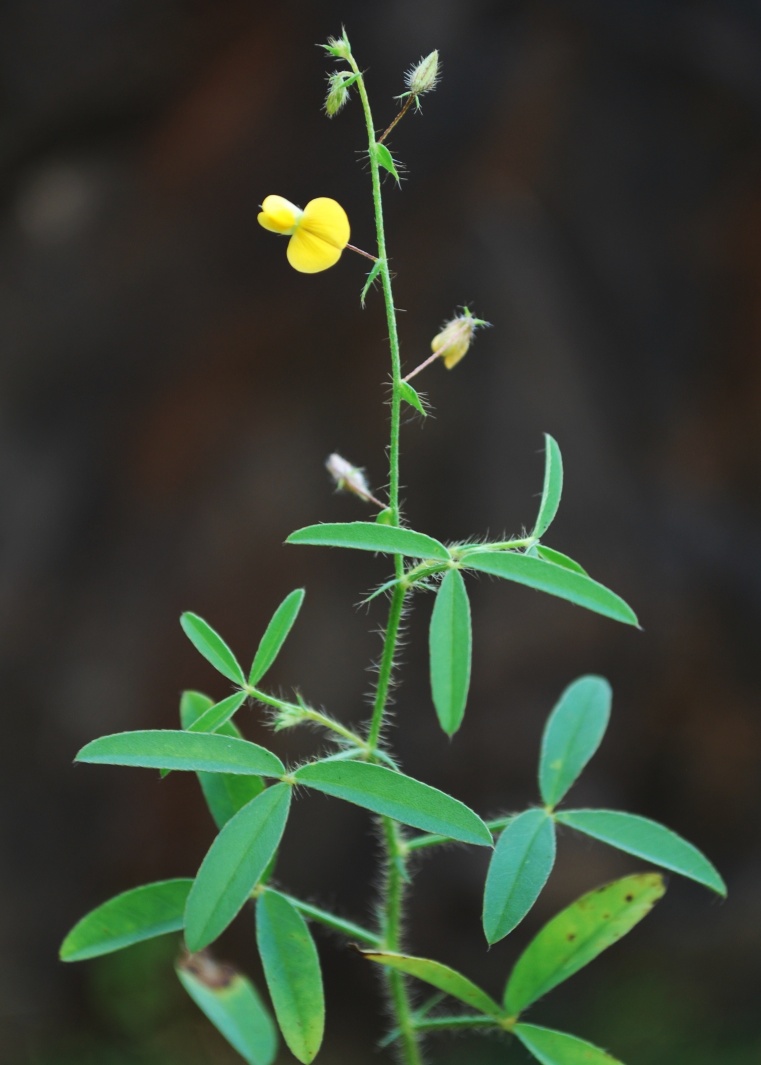
*Crotalaria orixensis* Willd.

**Figure 4a.**
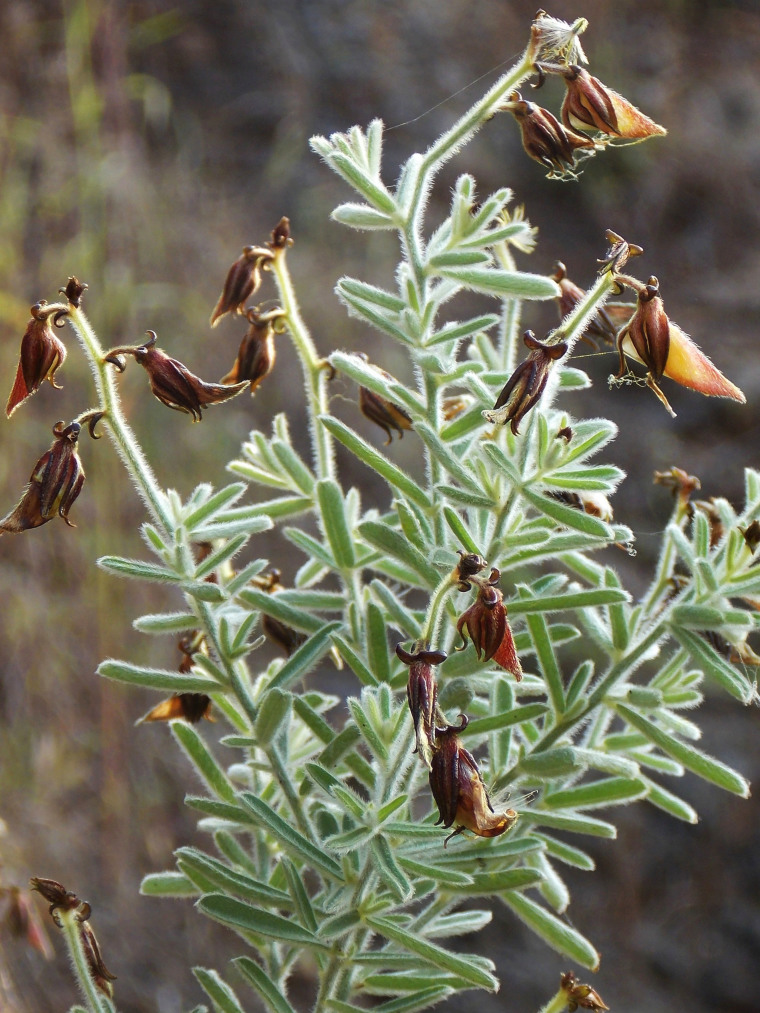
*Crotalaria ramosissima* Roxb.

**Figure 4b.**
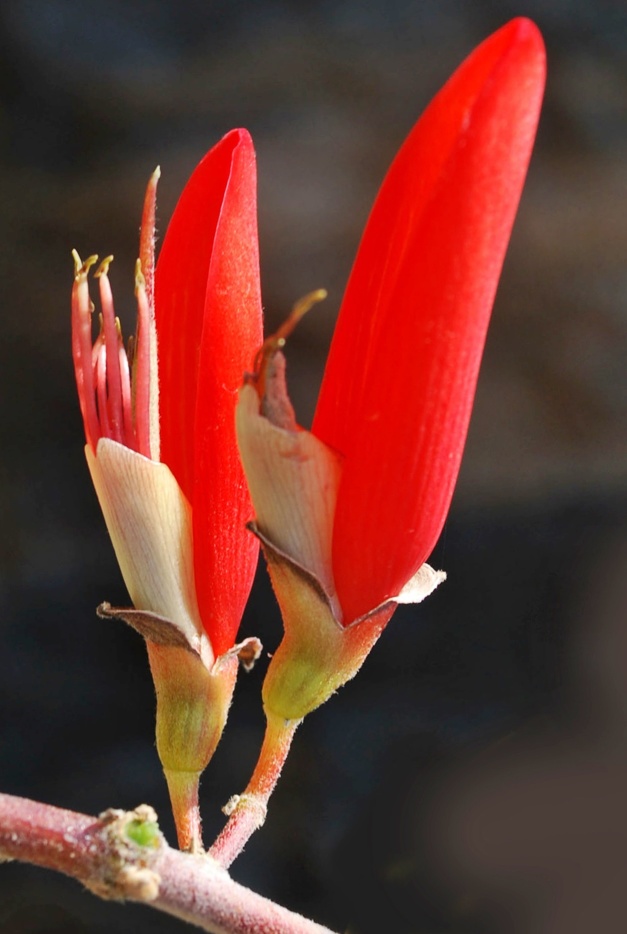
*Erythrina suberosa* Roxb.

**Figure 4c.**
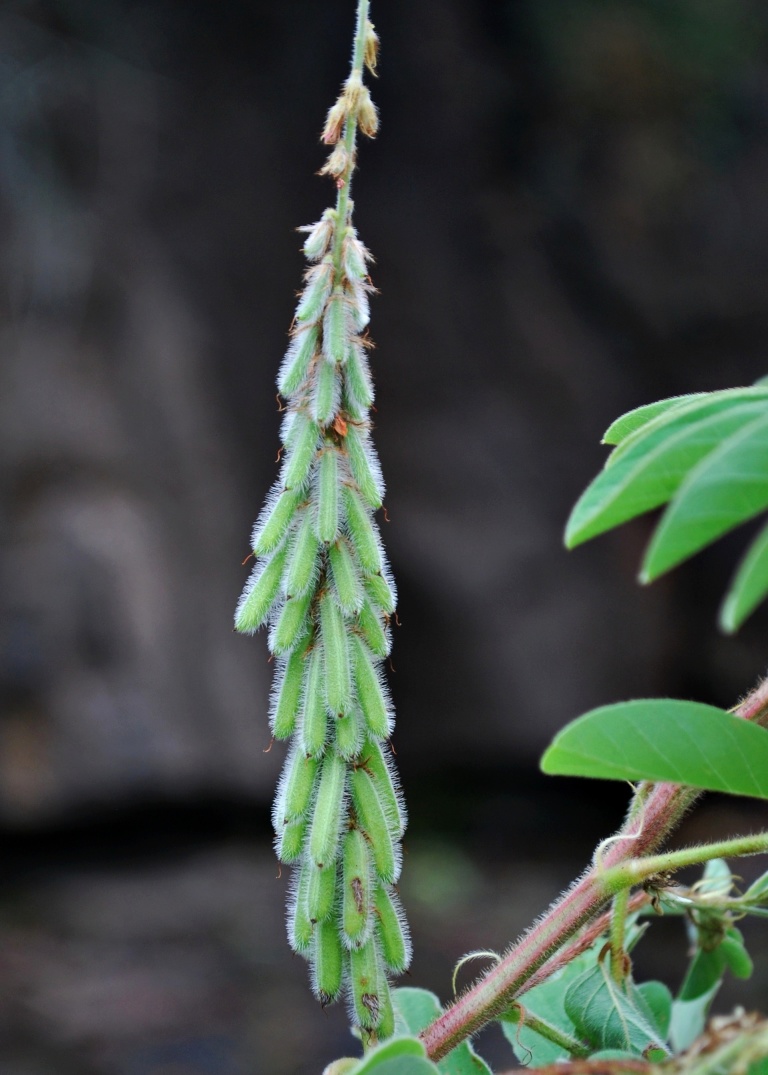
*Indigofera astragalina* DC.

**Figure 4d.**
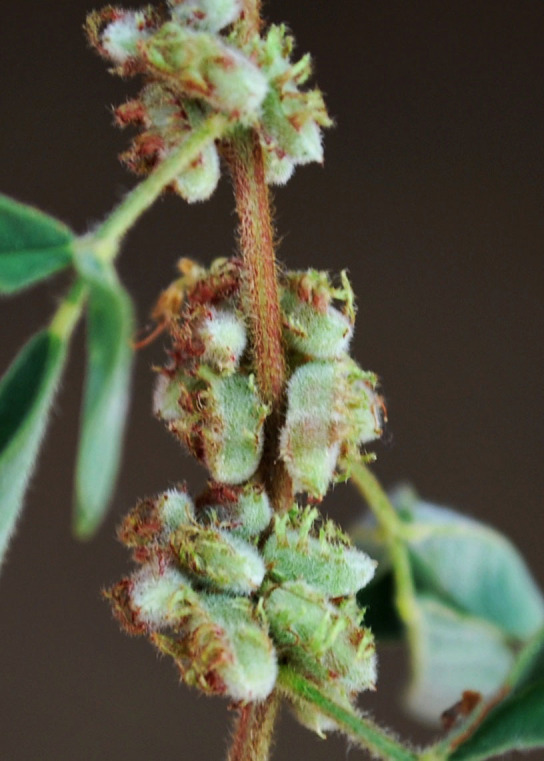
Indigofera glandulosa Wendl. var. glandulosa

**Figure 4e.**
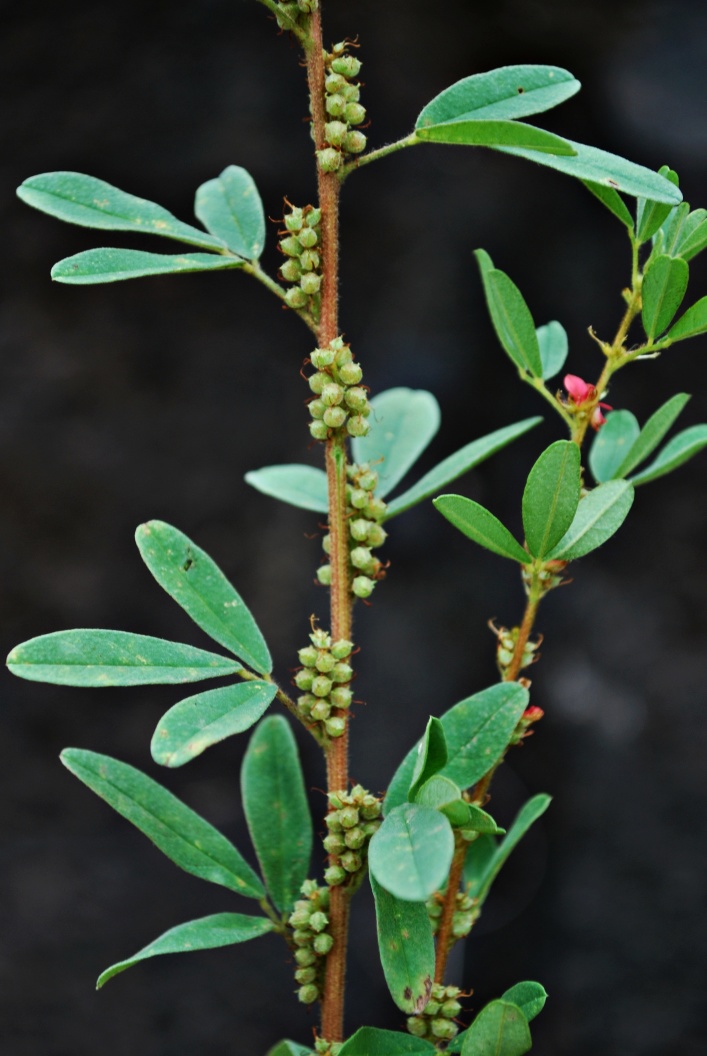
Indigofera glandulosa Wendl. var. sykesii Griff. ex Baker

**Figure 4f.**
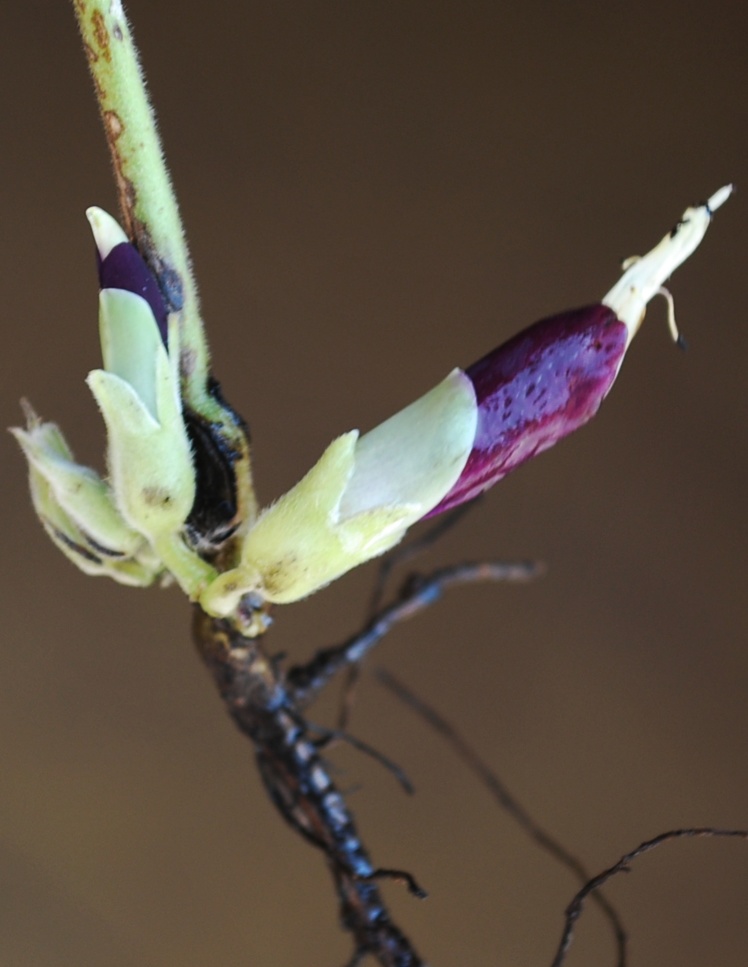
*Mucuna minima* Haines

**Figure 5a.**
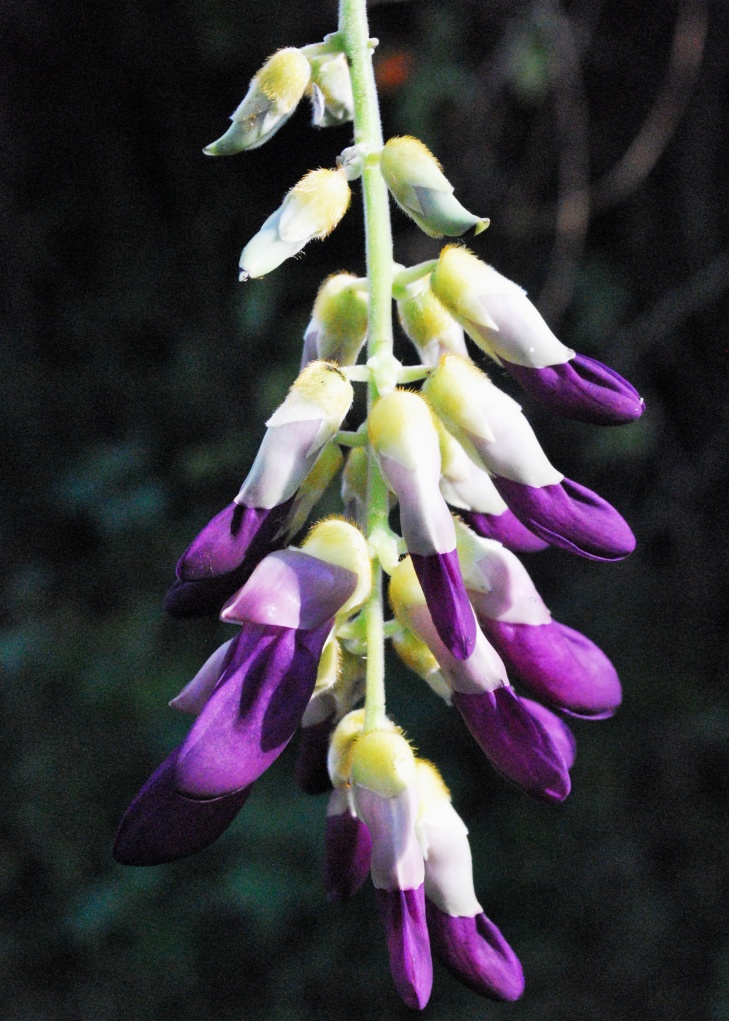
*Mucuna pruriens* (L.) DC.

**Figure 5b.**
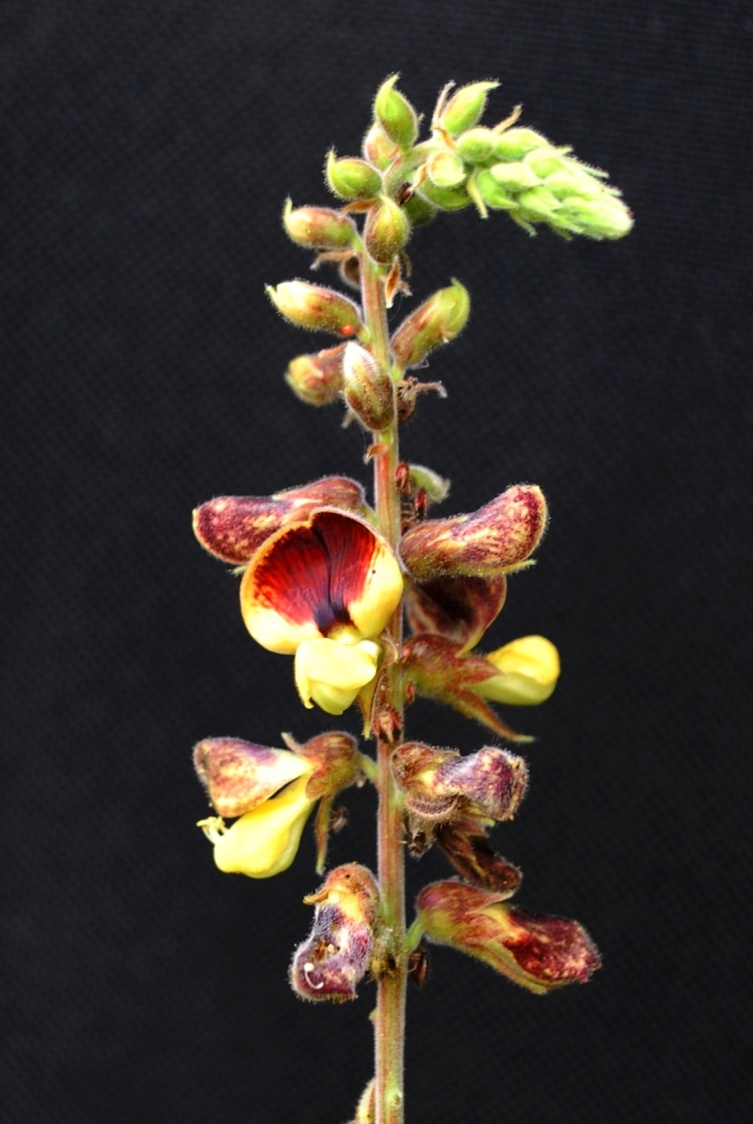
*Rhynchosia rothii* Benth. ex Ait.

**Table 1.** Leguminous Crop Wild Relatives (CWR) of Balaghat Ranges of Maharashtra. [*Note*:- **HR**- herbs; **SH**- shrubs; **THR**- trailing/twining herbs; **WCL**- woody climbers].

**Sr. No.**	**Name of the species**	**Habit**	**Type of crops**
1.	*Cajanus platycarpus* (Benth.) van der Maesen	THR	Pulses
2.	*Cajanus scarabaeoides* (L.) Du-Petit-Thours	THR	Pulses
3.	*Canavalia cathartica* Thours	WCL	Vegetable
4.	*Crotalaria juncea* L.	HR	Oil & fiber
5.	*Crotalaria leptostachya* Benth.	HR	Oil & Fiber
6.	*Crotalaria retusa* L.	SH	Oil & Fiber
7.	*Crotalaria verrucosa* L.	HR	Oil & fiber
8.	Lablab purpureus (L.) Sweet var. lignosus (L.) King	WCL	Vegetable
9.	Lablab purpureus (L.) Sweet var. purpureus Verdc.	WCL	Vegetable
10.	*Lens culinaris* Medik.	HR	Pulses
11.	*Stylosanthes fruticosa* (Retz.) Alston.	SH	Food & Fodder
12.	*Trigonella occulta* Del.	HR	Vegetable
13.	*Vigna aconitifolia* (Jacq.) Morchal.	THR	Pulses
14.	*Vigna indica* Dixit *et al.*	HR	Pulses
15.	*Vigna mungo* (L.) Hepper.	HR	Pulses
16.	*Vigna sublobata* (Roxb.) Babu & Sharma	THR	Pulses
17.	*Vigna trilobata* (L.) Verdc.	HR	Pulses
18.	*Vigna umbellata* (Thunb.) Ohwi & Ohashi	THR	Pulses
19.	Vigna unguiculata (L.) Walp. subsp. cylendrica (L.) Eselt.	THR	Pulses
